# Tryptophan Production Maximization in a Fed-Batch Bioreactor with Modified *E. coli* Cells, by Optimizing Its Operating Policy Based on an Extended Structured Cell Kinetic Model

**DOI:** 10.3390/bioengineering8120210

**Published:** 2021-12-10

**Authors:** Gheorghe Maria, Laura Renea

**Affiliations:** 1 Department of Chemical & Biochemical Engineering, University Politehnica of Bucharest, 011061 Bucharest, Romania; renea_laura@yahoo.com; 2 Chemical Sciences Section, Romanian Academy, Calea Victoriei 125, 010071 Bucharest, Romania

**Keywords:** glycolysis, tryptophan synthesis, modified *E. coli*, fed-batch bioreactor optimization, cell structured kinetic model, hybrid modular model

## Abstract

Hybrid kinetic models, linking structured cell metabolic processes to the dynamics of macroscopic variables of the bioreactor, are more and more used in engineering evaluations to derive more precise predictions of the process dynamics under variable operating conditions. Depending on the cell model complexity, such a math tool can be used to evaluate the metabolic fluxes in relation to the bioreactor operating conditions, thus suggesting ways to genetically modify the microorganism for certain purposes. Even if development of such an extended dynamic model requires more experimental and computational efforts, its use is advantageous. The approached probative example refers to a model simulating the dynamics of nanoscale variables from several pathways of the central carbon metabolism (CCM) of *Escherichia coli* cells, linked to the macroscopic state variables of a fed-batch bioreactor (FBR) used for the tryptophan (TRP) production. The used *E. coli* strain was modified to replace the PTS system for glucose (GLC) uptake with a more efficient one. The study presents multiple elements of novelty: (i) the experimentally validated modular model itself, and (ii) its efficiency in computationally deriving an optimal operation policy of the FBR.

## 1. Introduction

Over the last few decades, there has been a continuous trend to develop more and more effective bioreactors [1,2] “to industrialize important biosyntheses for producing fine chemicals used in the food, pharmaceutical, or detergent industry, by using free-suspended or immobilized cell cultures (or enzymes) in suitable bioreactors (or enzymatic reactors)”, as reviewed by Maria [3]. The batch (BR), semi-batch (fed-batch, FBR), a serial sequence of BRs [4], and the continuously operated fixed-bed or three-phase fluidized-bed bioreactors (with immobilized biocatalyst) are successfully used to conduct biosyntheses aimed at replacing complex chemical and energetically intensive processes, as well as those generating toxic wastes [5,6].

Applications include “fermentative processes for production of organic acids, alcohols, vinegar, amino acids/proteins, yeast, hydrogen, food products and additives, recombinant proteins/antibodies, etc., by using bioreactors with microbial (cell cultures) or enzymatic reactors [1,5] and by integrating genetic and engineering methods” [7,8].

Bioreactors with microbial/animal cell cultures have been developed in simple or complex constructive/operating alternatives as underlined in reviews by Maria [3] or by [9,10]. In spite of their larger volumes, the continuously mixing aerated tank reactors (CSTR), operated in BR or FBR modes, are preferred for bioprocesses requiring a high oxygen transfer and rigorous temperature/pH control. For these reasons, an effective FBR was used in the approached case study of TRP production.

From the engineering point of view, in addition to the production capacity optimization, there are several important issues to be addressed when screening among bioreactor alternatives and operating modes: (1) the maintenance of the bioprocess optimal conditions that ensure a high biomass activity (free or immobilized on a suitable porous support), by supporting its growth to compensate for “its natural biodegradation, and the risk to disintegrate the flocks or the support through mechanical shearing induced by the mixing, thus leading to the biomass leakage and washout”; (2) development of optimal operating policies based on an available process dynamic (kinetic) model derived from on/offline measurements. The model-based optimal operation of the bioreactor can be applied in two ways: (2a) “offline”, in which an optimal operating policy is determined on the basis of an adequate kinetic model (usually a deterministic one, based on the process mechanism), previously identified from separate experiments, and (2b) “online”, involving a simplified dynamic model identified using a classic state parameter estimator based on the online recorded data [11,12,13,14,15,16].

The current (default) approach to solve the model-based design, optimization, and control problems of industrial biological reactors is the use of unstructured models of Monod type (for cell culture reactors) or of Michaelis–Menten type (if only enzymatic reactions are retained), which ignores detailed representations of cell processes. The applied engineering rules are similar to those used for chemical processes and are inspired by the nonlinear system control theory [11,17,18,19,20,21,22,23,24,25,26]. However, by accounting for only key process variables (biomass, substrate and product concentrations), these models do not properly reflect the metabolic changes, being unsuitable to accurately predict the cell response to environmental perturbations by means of (self-)regulated cell metabolism.

The alternative is to use structured kinetic models, by accounting for cell metabolic reactions and component dynamics. Such deterministic models lead to a considerable improvement in the predictive power, at the expense of incorporating a larger number of species mass balances, including parameters (rate constants) difficult to be estimated from often incomplete cell data and, consequently, difficult to be used for industrial-scale purposes [27,28].

An alternative compromise is to use hybrid models that combine unstructured with structured process characteristics to generate more precise predictions [28,29,30,31,32]. The idea of hybrid kinetic models is to interconnect groups of process variables belonging to at least two hierarchical levels of model details. The resulting composite model is able to simulate the bioreactor dynamics simultaneously at various levels of detail. Thus, the dynamics of the bioreactor macroscopic state variables (i.e., species present in the liquid bulk) is simulated concomitantly to the nanoscale variables describing the cell metabolic processes of interest, because the macro/nanoscale variables are closely linked, as long as some cell metabolites are imported/excreted from/in the bioreactor bulk. Even if such a complex/extended dynamic model, including some complex cell metabolic pathways, requires more experimental and computational efforts to be built up and identified from structured kinetic data, the resulting hybrid (bilevel) dynamic model presents the following major and remarkable advantages: (i) the extended model allows further in silico (model-based) engineering developments (bioreactor design, offline optimization) of a higher accuracy compared to the unstructured/empirical models. For instance, such a hybrid model could better predict the optimal time stepwise continuously feeding policy of the FBR to increase the bioreactor production. This numerical analysis is approached here; (ii) the extended hybrid model can also be used for bioinformatics purposes, by evaluating the influence of the bioreactor operating conditions (control variables) on the dynamics of cell key species and metabolic fluxes involved in the synthesis of target metabolites [33,34,35]. Examples includes conditions for occurrence of glycolytic oscillations [36], oscillations in the TRP-operon expression [33,37], or conditions leading to a balanced cell growth (quasi-steady-state QSS, i.e., homeostasis [36]). All these in silico simulations can direct the design of genetically modified microorganisms (GMO) with desirable “motifs” [38]; (iii) the extended hybrid structured models can also be used to obtain lumped (reduced) dynamic models of the process useful for rapid engineering calculations/process control, by employing specific model reduction rules and a check in local operating domains (see the pioneering works of Villadsen and Nielsen [39], as well as the large number of subsequent contributions, such as [40,41,42,43] for nonlinear models or [44,45,46] for linear models). As a result, the bioprocess complexity may be described by a succession of local reduced models enfolded on the real process; (iv) as proven by several case studies, the hybrid bilevel structured models allow more robust extrapolation of the bioprocess behavior. For instance, Maria and Luta [28] optimized the mercury uptake by modified *E. coli* cells in an FBR; Maria et al. [38] optimized the succinate production by modified *E. coli* in batch mode; see also the reviews of Maria [3] or Dorka [47] on FBR optimization for mAbs production.

In fact, such a hybrid structured cell dynamic model must include only the essential parts of the central carbon metabolism (CCM), by incorporating the pathways responsible for the target metabolite synthesis and the lumped modules of the cell core, i.e., the glycolysis, the GLC uptake system (i.e., the phosphotransferase (PTS) or an equivalent system), the ATP recovery system, and others (if necessary in simulations); see, for instance, [33,34,38,48].

Special interest has been given to the accurate modeling of the glycolysis dynamics and its self-regulation [33,36,48,49], as most of the glycolysis intermediates are starting nodes for the internal production of several cell metabolites (e.g., amino acids, SUCC, CIT, TRP) [3,34,35,37,38].

This need to have good-quality structured cell models to simulate the dynamics (and regulation) of the bacteria CCM became a subject of very high interest over the last decades, allowing in silico design of GMO-s with desirable characteristics of various applications [31,32,50].

**Figure 1:**
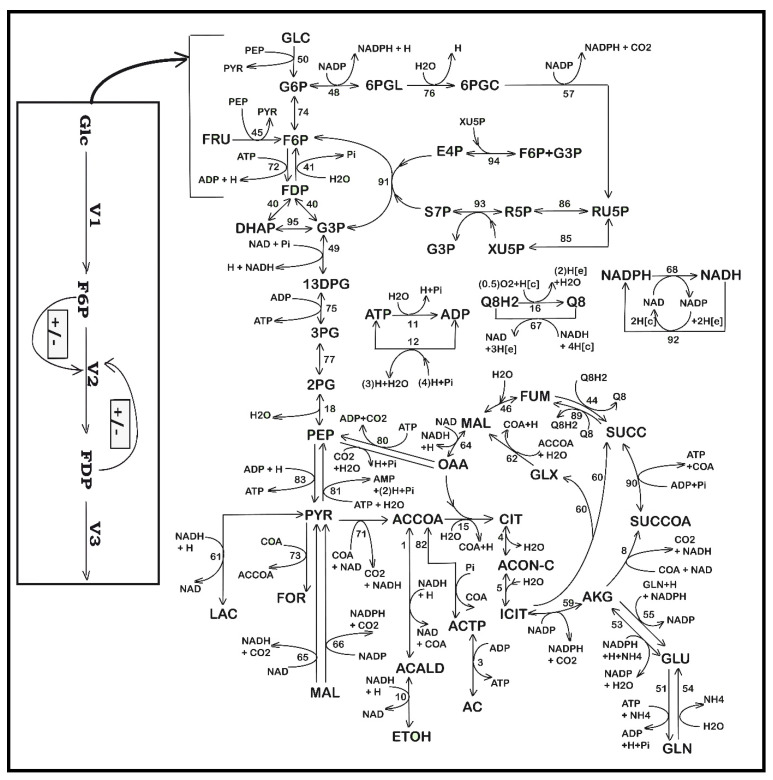
Simplified representation of the CCM pathway in *E. coli* of Edwards and Palsson [51] (i.e., the *wild* cell, including the PTS-system). Fluxes characterizing the membrane transport [*Metabolite*(e) ↔ *Metabolite*(c)] and the exchange with environment are omitted from the plot (see [38] for details and explanations regarding the numbered reactions). Notations: [e] = environment; [c] = cytosol. Adapted from Maria et al. [38] with the courtesy of CABEQ Jl. The considered 72 metabolites, the stoichiometry of the 95 numbered reactions, and the net fluxes for specified conditions are given by Maria et al. [38]. The left rectangle indicates the chemical node inducing glycolytic oscillations [33,36]. Notations [+] and [−] denote the feedback positive and negative regulatory loops, respectively. GLC = “glucose”. See the abbreviation list for species names; V1–V6 = lumped reaction rates discussed in the Section 3.1.3.

As a result, an impressive large number of valuable *structured deterministic* models (based on a mechanistic description of the metabolic enzymatic reactions tacking place among individual or lumped species) have been proposed in the literature to simulate the cell CCM dynamics, including tens to hundreds of key species. Here, it is worth mentioning the *E. coli* model of Edwards and Palsson [51] used by [38,48,52,53,54,55,56] for various purposes, the *S. cerevisiae* glycolysis model of Teusink et al. [57], the JWS platform of Olivier and Snoep [58], and the MPS platform of Seressiotis and Bailey [59] to simulate the cell metabolism (species dynamics, and/or fluxes), to mention but a few. Simulation platforms, such as E-cell [60,61] or V-cell [62], accounting for thousands of species and reactions, display extended capabilities to predict the dynamics of the cell metabolism under various conditions, based on EcoCyc, KEGG, Prodoric, Brenda, and other omics databanks (as reviewed by Maria [32]). Worthwhile CCM-based dynamic or stationary models were reported by Maria [35,38,48] and are schematically represented in (Figure 1). Meritorious structured deterministic kinetic models have been reviewed by Maria [31]. Deterministic kinetic models using continuous variables have been developed by Maria [48] for glycolysis, and by Schmid et al. [63], Chassagnole et al. [52], Costa et al. [64,65], and Machado et al. [66] for the CCM. Such models can adequately reproduce the cell response to continuous perturbations, with the cell model structure and size being adapted on the basis of available omics information. Even if such extended structured models are currently used only for research purposes, as they are difficult to be identified, it is a question of time until they will be adapted for industrial/engineering purposes in the form of reduced structured hybrid models. The case study discussed here proves the engineering aspect.

At this point, it is worth underlining that the cell metabolism is highly sophisticated, involving 10^3–4^ components, 10^3–4^ transcription factors (TF-s), activators, inhibitors, and at least one order of magnitude higher number of (bio)chemical reactions, all ensuring a fast adaptation of the cell to the changing environment through complex genetic regulatory circuits (GRC-s) [50]. The cell is highly responsive to the environmental stimuli and highly evolvable by self-changing its genome/proteome and metabolism, mediating the stoichiometry and the reaction rates (fluxes) of the enzymatic reactions to get an optimized and balanced growth by using minimum resources (nutrients/substrates).

Development of extended CCM dynamic models on a deterministic basis to adequately simulate *in detail* the cell metabolism self-regulation, cell growth, and replication for such an astronomical cell metabolism complexity is practically impossible due to the lack of structured and comprehensive information, as well as computational limitations. Reviews of some trials were presented by Styczynski and Stephanopoulos [67] and by Maria [31,32,50].

In spite of such tremendous modeling difficulties, the development of structured reduced deterministic (rather than stochastic) models [31] able to adequately reproduce the dynamics of some CCM complex metabolic syntheses [48,67,68], as well as the dynamics of the genetic regulatory systems [50] tightly controlling the metabolic processes, has reported significant progress over the last few decades [69,70]. Even if they are rather based on sparse information from various sources, unconventional statistical identification, and lumping algorithms [31,41,45,50], such structured reduced deterministic kinetic models have been proven to be extremely useful for in silico analysis and characterization of the CCM, as well as for the design of a novel GRC-s conferring new properties/functions to the mutant cells [31,50,71].

This paper is aimed at proving the feasibility and advantage of using this novel concept to couple an extended cell structured deterministic kinetic model with a bioreactor macroscopic dynamic model. The resulting hybrid dynamic model was successfully used for engineering evaluations. The applied example involves the optimization of the FBR used for TRP synthesis.

“l-tryptophan is a high-value aromatic amino acid with important applications in food and pharma industry. TRP is an aromatic nonpolar α-amino acid essential in humans, which is used in the cell biosynthesis of proteins, being also a precursor to the neurotransmitter serotonin, the melatonin hormone, and vitamin PP” [72].

This paper uses a hybrid dynamic model built up by Maria [35] by linking a CCM-based structured kinetic model with an FBR simple dynamic model. The resulting hybrid FBR model was used to computationally determine the optimal (time stepwise) feeding policy of the FBR used by Chen et al. [73] to study TRP synthesis using a modified *E. coli* T5 strain culture. The thus obtained optimal operating policy of the FBR has proven to be very effective, by ensuring maximization of TRP production involving a few key control variables (i.e., the feed flow-rate and the feeding GLC concentration), and it reported better performance compared to the non-optimally operated FBR of Maria et al. [34,35] or of Chen [74].

The structured modular kinetic model of Maria [35,48] used in this numerical analysis includes modules characterizing the dynamics of the concerned cell pathways involved in TRP synthesis, i.e., glycolysis, ATP recovery system, TRP operon expression, and biomass growth. This bioprocess model was experimentally identified and checked over extensive experiments conducted by several authors, i.e., [33,48,52,68] for glycolysis, and by Chen et al. [73,74] and Maria [35] for TRP synthesis. Experimental data of Chen [74] for TRP synthesis were also used to compare the derived predictions of the hybrid model.

The present study presents multiple elements of novelty: (i) although production of TRP by engineered *E. coli* has been extensively studied, “the need of multiple precursors for its synthesis and the complex regulations of the biosynthetic pathways make the achievement of a high product yield still very challenging” [35]. This engineering problem was solved here by using a model-based (in silico) approach, completed with a biological improvement of the used *E. coli* cell culture; (ii) the derived optimal operating policy of the FBR is given in time intervals (the so-called “time-arcs”) of equal length, with a reduced number, to be easily implemented. The control variables present optimal but constant levels over each time-arc (different between time-arcs) during the FBR operation; (iii) the used biomass culture refers to a modified *E. coli* T5 strain. The characteristics of this strain were reflected in the rate constants estimated by Maria [35]. This T5 strain was produced by Chen [75] and Chen et al. [73] to increase TRP production in their bench-scale FBR. They performed genetic modifications of the TRP-producing “wild” strain S028. Basically, “they removed the PTS import-system of GLC, by replacing it with a more effective one based on the galactose permease/glucokinase (GalP/Glk) uptake system, by modulating the gene expression of GalP/Glk. The resulting T5 strain showed an increase of the specific TRP production rate in a nonoptimal FBR by 52.93% (25.3 mg/gDW biomass /h) compared to the initial strain” [73] and by ca. 70% if the used FBR was optimally operated (this paper); (iv) the results reveal the close link between the cell key metabolites and the FBR operating conditions; (v) the used hybrid bilevel kinetic model is complex enough to adequately represent the dynamics of the FBR state variables (i.e., the biomass growth, the GLC depletion, and the excreted TRP and PYR in the bulk phase), as well as the dynamics of the cell key species involved in the concerned reaction pathway modules, i.e., (a) glycolysis, (b) ATP recovery system, and (c) TRP operon expression.

## 2. *E. coli* T5 Strain and the Experimental FBR

### 2.1. The Used E. coli Strain

**Figure 2:**
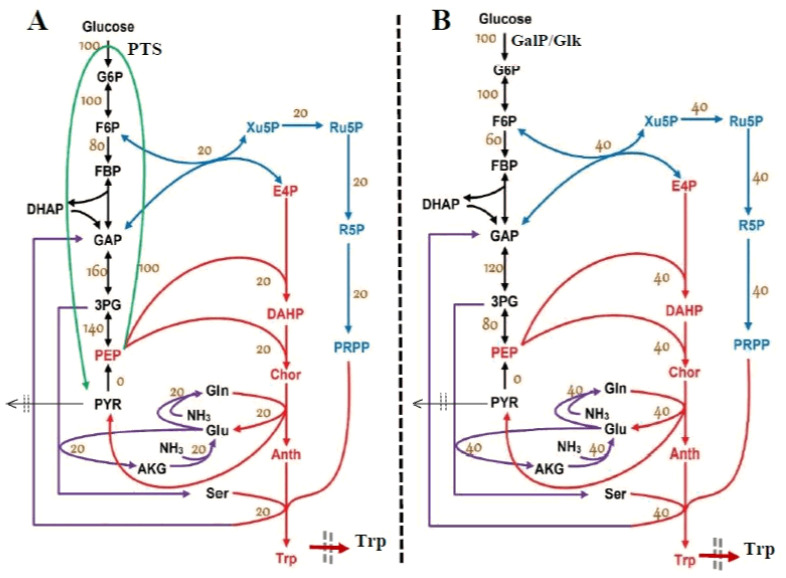
Comparison between the reduced schemes for GLC import systems into the cell linked to the TRP synthesis. Adapted from [73,74] (see the acknowledgement). (**A**) The wild *E. coli* model of Chassagnole et al. [52] and Maria [48] uses the phosphoenolpyruvate/sugar phosphotransferase (PTS) system for the GLC uptake. (**B**) The modified *E. coli* T5 strain of Chen et al. [73] and Chen [74], studied in this paper, uses the more efficient GLC uptake system based on galactose permease/glucokinase (GalP/Glk). The numbers on arrows indicated the relative metabolic fluxes at QSS predicted by Chen [74,75]. The same authors predicted a maximum theoretical yield of 0.23 g Trp/g glucose for the wild *E. coli* strain and of 0.45 g Trp/g glucose for the modified T5 strain.

Although production of TRP by engineered *E. coli* has been extensively studied, the need of multiple precursors for its synthesis, and the complex regulations of the biosynthetic pathways make the achievement of a high product yield still very challenging. The metabolic flux analysis [74,75,76] suggests that replacement of the PTS glucose uptake system in the wild *E. coli* with the galactose permease/glucokinase (GalP/Glk) uptake system can double the TRP yield from glucose. These authors obtained a promising *E. coli* T5 strain which, tested in an FBR, showed an increased GLC import capacity together with an increased TRP yield by ca. 20% compared to an initial mutant S028 strain (i.e., 0.164 vs. 0.137 g TRP/g GLC), while the specific production rate was increased by 53% [73]. The cell flux analysis by Chen [74,75] indicated (Figure 2) the doubling of fluxes responsive to TRP synthesis. Finally, a highly productive strain T5AA was obtained, with a TRP production rate of 28.83 mg/gDW/h [73,74,76,77]. More details on *E. coli* mutants presenting alternative routes for GLC uptake were given by Chen et al. [73], Chen et al. [77], Chen and Zeng [76], Chen [74], Li et al. [78], Niu et al. [79], and Carmona et al. [80].

### 2.2. Experimental Bioreactor and the Recorded Kinetic Data

**Figure 3:**
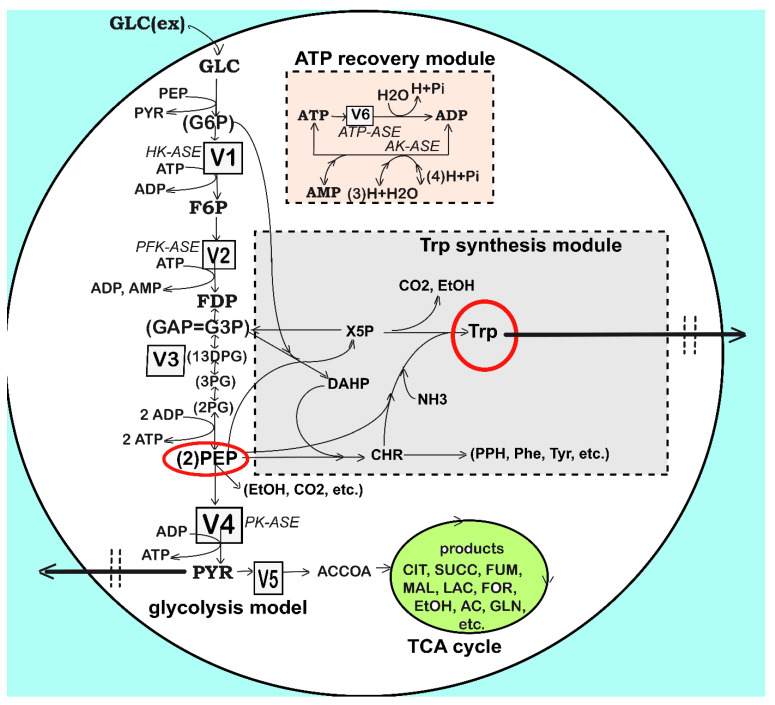
Simplified structured reaction pathway in *E. coli* for glycolysis (after Maria [48]), and for the TRP synthesis (the gray area) (after Maria et al. [35,37,48]). This reaction pathway was used by Maria et al. [34,37] to derive a TRP synthesis kinetic model. Connection of the TRP synthesis to glycolysis is realized through the PEP node [33,37]. The modular model structure also includes the synthesis of adenosine cometabolites ATP, ADP, and AMP, as part of the ATP recovery system (the pink rectangle in the figure). Notations: GLC(ex)= glucose in the cell environment. Species abbreviations are given in the abbreviations list. Species in parenthesis are not explicitly included in the glycolysis model. Italic letters denote the enzymes. Squares include notations of enzymatic reactions V1–V6 included in the glycolysis model (Table 2 and Table 3). Adapted from [48] with the courtesy of CABEQ Jl., and completed according to the Maria [35] kinetic model.

**Figure 4:**
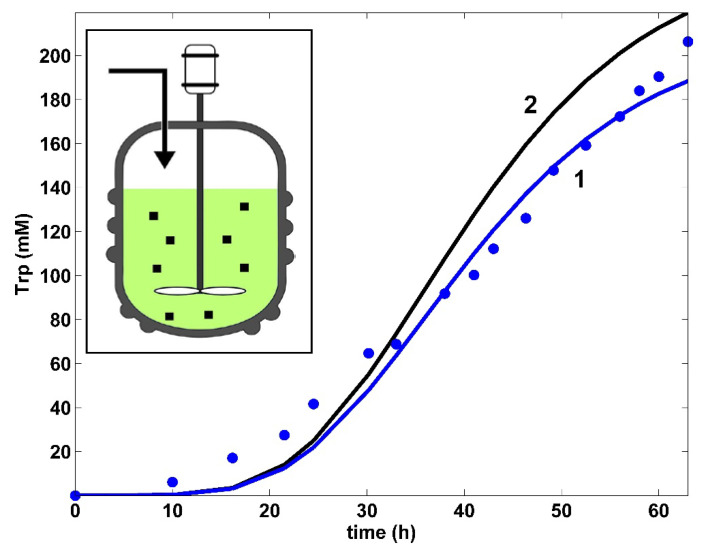
Model-based predictions of the tryptophan (Trp) concentration dynamics in the same FBR using the modified *E. coli* T5 strain, but operated in two alternatives: (i) (2, black) optimal operation derived in this paper (i.e., variable fed [GLC] and variable feed flow rate), or (ii) (1, blue) simulations [35] and the experimental data (●, blue) of Chen [74] for the nominal, nonoptimal operation of Table 1, with a constant fed [GLC] and a constant feed flow rate. (Left corner) A simplified scheme of the used FBR with suspended biomass (small points).

To estimate the rate constants of the hybrid structured kinetic model for the studied TRP synthesis using the modified *E. coli* T5 strain (Figure 3), Maria [35] used the experimental kinetic data of Chen [74] obtained in a lab-scale three-phase FBR operated under the so-called “nominal” (nonoptimal) conditions displayed in (Table 1). The completely automated FBR of 1.5 L capacity includes a large number of facilities described in detail by Chen [74]. The nominal operation of this bioreactor by Chen [74] requires addition of a controlled constant feed flow rate of substrate solution (GLC) of a constant concentration, together with nutrients, antibodies, etc. in recommended amounts (Table 1) along the entire batch. A reduced FBR scheme can be found in the upper left corner of Figure 4.

**Figure 5:**
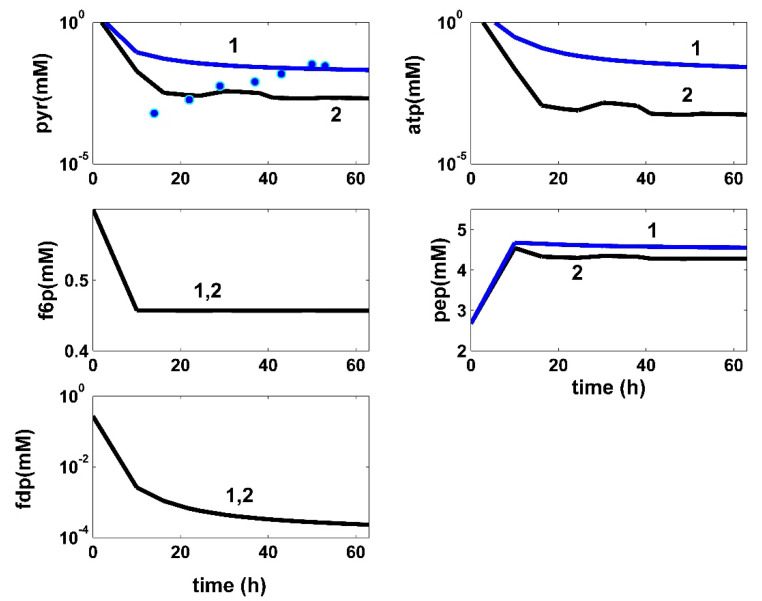
Model-based simulated trajectories (-) for the glycolytic key species (PYR, F6P, FDP, ATP, and PEP) in the modified *E. coli* T5 strain for the FBR operated in two alternatives: (i) (2, black) optimal operation derived in this paper (variable fed [GLC] and variable feed flow rate), and (ii) (1, blue) the experimental data (●, blue) of Chen [71] recorded under nominal, nonoptimal operation of Table 1, with a constant fed [GLC] and a constant feed flow rate. Species abbreviations are given in the abbreviations list.

**Figure 6:**
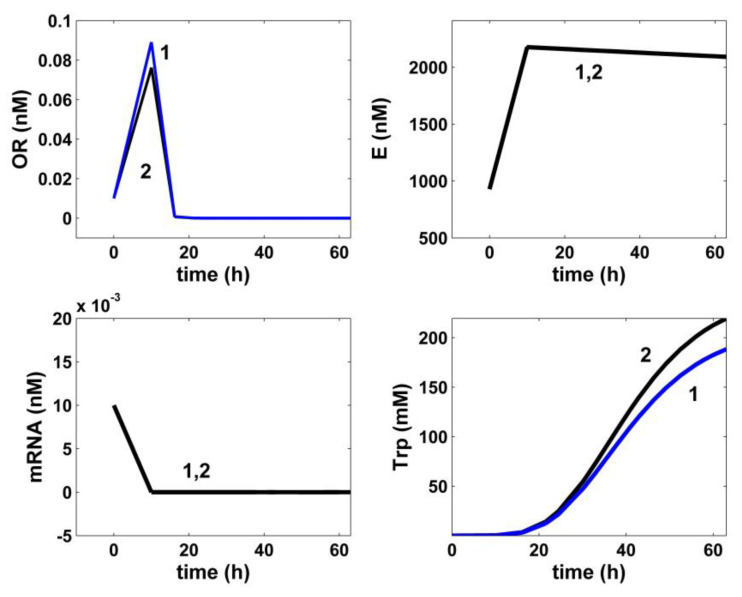
Model-based simulated trajectories (-) for the key species involved in the TRP operon expression module (TRP, OR, mRNA, and E) in the modified *E. coli* T5 strain for the FBR operated in two alternatives: (i) (2, black) optimal operation derived in this paper (variable fed [GLC] and variable feed flow rate), and (ii) (1, blue) under nominal, nonoptimal operation of Table 1, with a constant fed [GLC] and a constant feed flow-rate. Species abbreviations are given in the abbreviations list.

**Figure 7:**
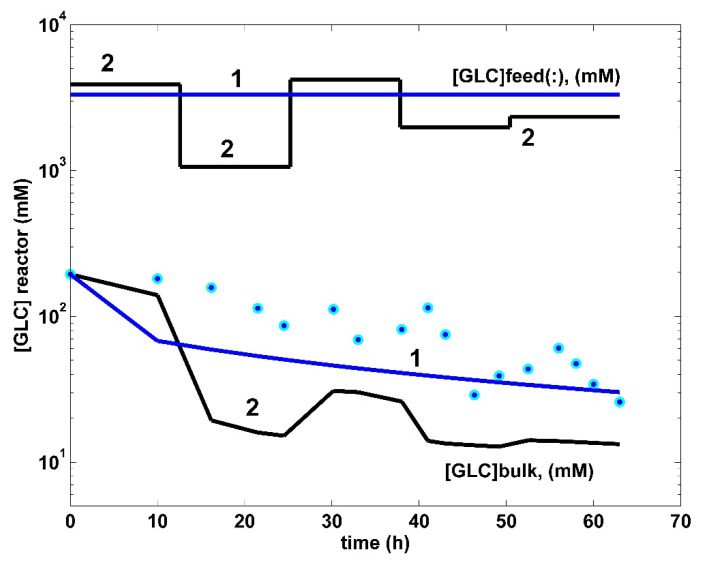
Top curves. The time stepwise optimal feeding policy (2, black) of the GLC concentration in the bioreactor $c_{glc,j}^{feed}$ (*j* = 1, …, 5 time-arcs), derived in this paper (variable fed [GLC] and variable feed flow rate). Comparison is made with the experimental FBR (1, blue) operated under the nominal (nonoptimal) operating conditions of Table 1, with a constant feed flow rate, and with a constant GLC concentration in the feed. Both cases use the same modified *E. coli* T5 strain. (Bottom curves). Model-based simulated trajectories (—) of glucose (GLC) in the bioreactor bulk for the FBR operated in two alternatives: (i) (2, black) optimal operation derived in this paper (variable fed [GLC] and variable feed flow rate), and (ii) (1, blue) experimental data (●, blue) of Chen [71] derived under nominal, nonoptimal operation of Table 1, with a constant fed [GLC] and a constant feed flow rate.

**Figure 8:**
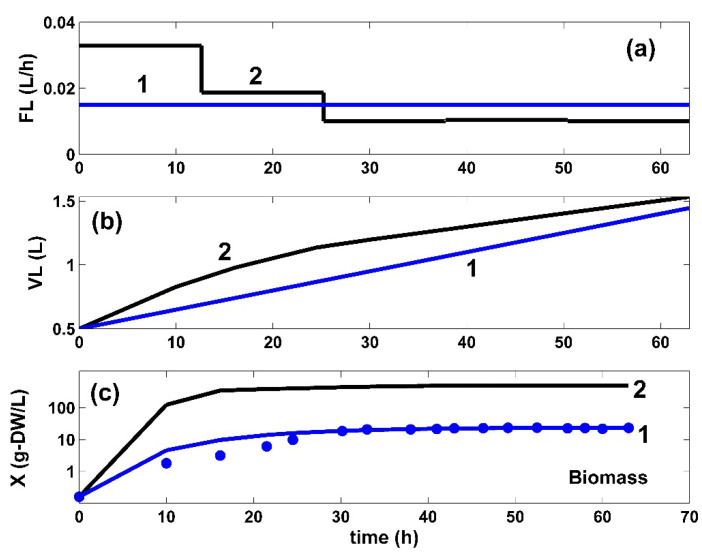
(**a**). The time stepwise optimal policy of the feed flow-rate (FL), (*j* = 1, …, 5 time-arcs) in the bioreactor (—) for the FBR operated in two alternatives: (i) (2, black) optimal operation derived in this paper (variable fed [GLC] and variable feed flow rate), and (ii) (1, blue) trajectories under nominal, nonoptimal operation of (Table 1), with a constant fed [GLC] and a constant feed flow rate. Both cases use the same modified *E. coli* T5 strain. (**b**) The liquid volume (VL) dynamics in two alternatives: (i) using the optimal policy of the variable feed flow rate (FL) in the bioreactor (2, black) derived in this paper, or (ii) using (1, blue) the nonoptimally operated FBR under the nominal conditions of Table 1, with a constant fed [GLC] and a constant feed flow rate. (**c**). The model-based predictions of the biomass (X) concentration in the same FBR with using the modified *E. coli* T5 strain, but operated in two alternatives: (i) (2, black) optimal operation derived in this paper (i.e., variable fed [GLC] and variable feed flow rate), or (ii) (1, blue) simulations and the experimental data (•, blue) of Chen [71] under nominal, nonoptimal operation of Table 1, with a constant fed [GLC] and a constant feed flow rate.

To obtain the necessary kinetic data, samples were taken from the FBR bulk during the batch (63 h), with a certain frequency (2 to 5 h), thus determining the concentration dynamics of the key species of interest, i.e., X (biomass), GLC, TRP, and PYR. These recorded data are presented in Table 1 (see also the blue points in Figure 4, Figure 5, Figure 6, Figure 7 and Figure 8). The reader interested in the analytical methods used to obtain the experimental data, as well as in the details related to the bench-scale bioreactor operation and to the data acquisition system, is referred to the PhD thesis of Chen [74] (see also the Acknowledgement).

**Table 1:** The nominal initial operating conditions of the FBR used by Chen [74] to collect the kinetic data of the TRP synthesis using a suspended culture of genetically modified *E. coli* cells (T5 strain). More experimental details were given by Chen [74].

The FBR Initial Conditions		
Parameter	Nominal (Initial) Value	Obs.
Bioreactor type	DASGIP parallel bioreactor system, Eppendorf (completely automated)	[74]
Bioreactor mixing	Both mechanical and sparkling gas (O_2_)	[74]
Oxygen supply	Pure oxygen sparging	[74]
$\mathrm{Biomass}\;\mathrm{initial}\;\mathrm{concentration}\;(c_{x}{}_{,0}$) (g DW·L^−1^)	0.16 Experimental data of Chen [74] (Figure 8c)	With the courtesy of Chen [74]
Batch time (*t_f_* )	3780 min (63 h)	
Cell content dilution rate (μ), (1/min)	1.25 × 10^−5^–0.015	Estimated 0.0017 [52]
$\mathrm{Feed}\;\mathrm{flow}\;\mathrm{rate}\;(F_{L}$)	0.015 L h^−1^	Maintained quasi-constant
$\mathrm{Bioreactor}\;\mathrm{liquid}\;\mathrm{initial}\;\mathrm{volume}\;(V_{L,0}$)	0.5 L (initial)	Variable, due to the continuous feeding of the FBR
$\mathrm{Glucose}\;\mathrm{feeding}\;\mathrm{solution}\;\mathrm{concentration}\;c_{glc}^{feed}$	3330.5 mM	Maintained constant by Chen [74]
$\mathrm{Initial}\;\mathrm{glucose}\;\mathrm{concentration}\;\mathrm{in}\;\mathrm{the}\;\mathrm{bioreactor}\;c_{glc}^{ext}$ at (*t* = 0)	194.53 mM Experimental data of Chen [74] (Figure 7)	[74]
Temperature/pH	37 °C/6.8	[74]
$\mathrm{Bioreactor}\;\mathrm{capacity}\;[\max(V_{L}$)], and facilities	3 L, automatic control of pH, DO, temperature	[74]
$\mathrm{Biomass}\;\mathrm{density}\;(\rho_{x}$)	565.5 g DW·L cytosol^−1^	[52]
Initial concentrations for the glycolytic cell species (in mM)	$c_{F6P}(t=0)$ = 0.6003 $c_{FDP}(t=0)$ = 0.2729 $c_{PEP}(t=0)$ = 2.6729 $c_{PYR}(t=0)$ = 2.6706 $c_{ATP}(t=0)$= 4.27 [AMDTP]total = 5.82	Measured by Chassagnole et al. [52]
Initial concentrations for the TRP synthesis operon species (in μM)	$c_{OR}$ (*t* = 0) = 0.01 $c_{OT}$ (*t* = 0) = 3.32 (nM) $c_{MRNA}$ (*t* = 0) = 0.01 $c_{E}$(*t* = 0) = 928 (nM)	Measured by Bhartiya et al. [81]
	$c_{TRP}$(*t* = 0) = 0.164	This paper; data of Chen [74]

**Table 2:** Mass balance of the cell glycolytic key species and of the FBR control variables (GLC, *F_L_*) for the optimally operated (time stepwise feeding policy) FBR, adapted from Maria [33,34,35,37,48].

Species Mass Balance	Auxiliary Relationships and Estimated Rate Constants
Glucose $\frac{dc_{glc}^{ext}}{dt}=\frac{F_{L,j}}{V_{L}(t)}\left(c_{glc,j}^{feed}-c_{glc}^{ext}\right)-\frac{c_{x(t)}}{\rho_{x}}V_{1}$ $c_{glc,j}^{feed}$ = control variables to be optimized; *j* = 1,…, $N_{div}$ (equal time-arcs) $c_{glc}^{ext}$ (*t* = 0) is given in (Table 1) for the nominal FBR of Chen [74] For the optimal FBR with adopted $N_{div}$ = 5, the feeding policy is (Footnote a): $c_{glc,j}^{feed}=\{\begin{array}{c} c_{glc,0}^{feed}\;\mathrm{if}\;0\;\leq t\;<\;T1\;\\ c_{glc,1}^{feed}\;\mathrm{if}\;T1\;\leq t\;<\;T2\\ c_{glc,2}^{feed}\;\mathrm{if}\;T2\;\leq t\;<\;T3\\ c_{glc,3}^{feed}\;\mathrm{if}\;T3\;\leq t\;<\;T4\\ c_{glc,4}^{feed}\;\mathrm{if}\;T4\;\leq t\;<\;t_{f} \end{array}$	$(i)\;c_{amp}+c_{adp}+c_{atp}=c_{amdtp}$ = constant [33,86,87] (ii) $c_{adp}$ results from solving the thermodynamic equilibrium relationship $c_{atp}c_{amp}=Kc_{adp}^{2}$ , i.e., $c_{adp}^{2}\frac{K}{c_{atp}}+c_{adp}-c_{amdtp}+c_{atp}=0$ (iii) μ = cell dilution rate (Table 1) (iv) The initial values of cell species concentrations are given in Table 1 (see also footnote (b)) (v) The lump $c_{tca}$ of Figure 3 includes species belonging to the TCA cycle; there are no measurements on this lump, so it was excluded from data fitting (vi) The adopted value for $y_{trp}$ by Maria [35] is $y_{trp}=r_{syn,trp}/r_{syn,pep}$ = 1/43.63 (at QSS) [88]; $y_{trp}$ was re-estimated from experimental data by Maria [35], resulting in $y_{trp}$ = 0.467 (vii) See Table 3 for the $V_{1}\;-\;V_{6}$ flux expressions
Species inside the cell $\frac{dc_{f6p}}{dt}=V_{1}-V_{2}-\mu \;c_{f6p}$	
$\frac{dc_{fdp}}{dt}=V_{2}-V_{3}-\mu \;c_{fdp}$	
$\frac{dc_{pep}}{dt}=2\;V_{3}-V_{4}-y_{trp}\left(2\;V_{3}\right)\;-\;\mu \;c_{pep}$	
$\frac{dc_{pyr}}{dt}=V_{4}-V_{5}-\mu \;c_{pyr}$ $\;\frac{dc_{atp}}{dt}=-V_{1}-V_{2}+2\;V_{3}+V_{4}-V_{6}-\mu \;c_{atp}$	
Liquid volume dynamics $\frac{d\;V_{L}}{dt}=F_{L,j}$$;\;V_{L}\left(t=0\right)=V_{L,0}$ in Table 1; *j* = 1,…, $N_{div}$(equal time-arcs)	(viii) For the adopted $N_{div}$ = 5, the feeding policy is (see footnote (a)) $F_{L,j}=\{\begin{array}{c} F_{L,0}\;\mathrm{if}\;0\;\leq t\;<\;T1\;\\ F_{L,1}\;\mathrm{if}\;T1\;\leq t\;<\;T2\\ F_{L,2}\;\mathrm{if}\;T2\;\leq t\;<\;T3\\ F_{L,3}\;\mathrm{if}\;T3\;\leq t\;<\;T4\\ F_{L,4}\;\mathrm{if}\;T4\;\leq t\;<\;t_{f} \end{array}$
Biomass dynamics $\frac{dc_{x}}{dt}=\frac{\mu_{x}\;c_{glc}\;c_{x}}{{\left(a_{x}\;\exp\left(b_{x}\;t\right)\right)}^{N_{x}}}$; $c_{x}\left(t=0\right)=c_{x}{}_{,0}$ in (Table 1)	(ix) The biomass growth inhibition corresponds to a modified Contois model [85] The estimated rate constants by Maria [35] are $\mu_{x}$ = 1.05·10^−4^ (1/min·mM), $a_{x}$ = 10.19, $b_{x}$ = 1.8036·10^−2^ (1/min), $N_{x}$= 7.334 × 10^−2^

(a) For the adopted $N_{div}$ = 5, *j* = 1, …, the $N_{div}$ time-arc approximate switching points are T1 = 12.5 h, T2 = 25 h, T3 = 37.5 h, T4 = 50 h, $\;t_{f}$ = 63 h. The $F_{L,0}\;-\;F_{L,4}$ time stepwise feed flow rates are determined together with the other control variables (i.e., $c_{glc,j}^{feed}$) to ensure the FBR optimal operation. (b) The initial concentrations of cell species (F6P, FDP, PEP, PYR, ATP) and of the biomass are given in Table 1.

**Table 3:** Reaction rate expressions V1–V6 of the hybrid model of Table 2, describing the dynamics of the cellular glycolytic species according to the kinetic model of Maria [35,48] and of Chassagnole et al. [52]. In the present study, this glycolysis kinetic model was modified by replacing the PTS system (V1 flux) for the GLC uptake with those of the mutant T5 *E. Coli* strain tested in this paper. The model rate constants were estimated by Maria [46] to fit the experimental data of Chen [71] (presented in Table 1 and Figure 4, Figure 5, Figure 6, Figure 7 and Figure 8). Species abbreviations are given in the abbreviation list.

Reactions	Rate Expressions	Estimated Rate Constants (Units in mM, min)
GLC import system glc + pep → f6p + pyr pyr + atp → pep + adp + h glc + atp → f6p + adp +h	Modification for the T5 strain $V_{1}\;=\;r_{uptake}\;=\;\rho_{x}/c_{x}\;\cdot$ $\frac{r_{uptake}^{\max}c_{glc}^{ext}}{\left(K_{PTS,a1}+c_{glc}^{ext}\right)}$	$r_{uptake}^{\max}$ = 1.1191 (1/min) $K_{PTS,a1}$ = 3487.5 (mM) $K_{PTS,a2}$ = 0 $K_{PTS,a3}$ = 0
f6p + atp → fdp + adp + h	$V_{2}=r_{PFK}=\frac{(V_{1}/V_{2m})\;c_{f6p}^{\delta }}{\left(K_{2m}^{\delta }+K_{2m}^{\delta }{\left[\frac{K_{R}^{amp}}{K_{T}^{atp}}\right]}^{n}{\left(\frac{c_{atp}}{c_{amp}}\right)}^{n}+c_{f6p}^{\delta }\right)}$	δ = 1.0437 n = 2 $V_{2m}$ = 0.062028 (mM/min) $K_{2m}$= 6.16423 (mM) $K_{R}^{amp}$= 25 μM $K_{T}^{atp}$= 60 μM
fdp + 2 adp (+ 2 nad + 2 p) ↔ 2 pep + 2 atp (+ 2 nadh + 2 h + 2 h2o)	$V_{3}=k_{3}c_{fdp}^{\alpha }-k_{3p}c_{pep}^{\beta }$	$k_{3}$ = 4602.3 (1/min) $k_{3p}$ = 31.917 (1/min) α= 0.05 β= 3
pep + adp + h → pyr + atp	$V_{4}{=r}_{\mathrm{PK}}=\frac{(V_{1}/V_{4m})c_{pep}^{\gamma }}{\left(K_{4m}^{\gamma }+K_{4m}^{\gamma }{\left[\frac{K_{R}^{fdp}}{K_{T,PK}^{atp}}\right]}^{m}{\left(\frac{c_{atp}}{c_{fdp}}\right)}^{m}+c_{pep}^{\gamma }\right)}$	γ = 1.331879 *m =* 4 $V_{4m}$ = 0.1333655 (mM/min) $K_{4m}$= 1.146443 (mM) $K_{R}^{fdp}$= 0.2 (mM) $K_{T,PK}^{atp}$ = 9.3 (mM)
pyr → products (accoa, cit, succ, lac, etoh, ac, …)	$V_{5}=\frac{k_{5}c_{pyr}^{n_{consum,pyr}}}{K_{\mathrm{consum},\mathrm{pyr}}+c_{pyr}}$	$k_{5}$= 693.3544 (1/min) $K_{\mathrm{consum},\mathrm{pyr}}$ = 395.525 (mM) $n_{consum,pyr}$= 2.6814
atp → adp +h	$V_{6}=k_{6}c_{atp}$	$k_{6}$= 552.38 (1/min)
2 adp ↔ atp + amp	$c_{atp}c_{amp}=Kc_{adp}^{2}$	*K* = 1
	(i) Termonia and Ross [86,87] indicated experimental evidence of a very fast reversible reaction catalyzed by *AKase*, with the equilibrium being quickly reached (ii) The k6 constant takes values according to the microorganism phenotype (related to the gene encoding the enzyme *ATPase* that catalyzes this reaction) (iii) $c_{amp}+c_{adp}+c_{atp}=c_{amdtp}$ = constant [86,87] (iv) $c_{adp}$ results from solving the following thermodynamic equilibrium relationship: $c_{atp}c_{amp}=Kc_{adp}^{2}$, i.e., $c_{adp}^{2}\frac{K}{c_{atp}}+c_{adp}-c_{amdtp}+c_{atp}=0$.	

## 3. Bioprocess and Bioreactor Dynamic Model

### 3.1. The Structured Hybrid Kinetic Model of Maria

Being a metabolite of high practical importance, intense efforts have been invested to decipher the synthesis regulation mechanism of TRP in various microorganisms, for deriving an adequate dynamic model of its QSS or oscillatory synthesis to be used for engineering purposes. Some results include the deterministic kinetic models of [37,81], while other studies [63] rather focused on determining correlations among flux distribution, flux control, and the optimized enzyme amount distribution, by employing a reduced kinetic model, not able to simulate most CCM reaction pathways.

As the TRP synthesis regulation is a very complex process, a significant number of simplified kinetic models with lumped terms (species and/or reactions) have been proposed in the literature (see the review of Maria et al. [34,37]). Kinetic modeling of this complex process is even more difficult because, as proven by Xiu et al. [82,83], Chen et al. [84], and Maria [33,36,37], under certain FBR operating conditions, TRP synthesis can become an oscillatory process. Oscillations in the TRP synthesis are produced due to the concomitant activation and high-order repression of the TRP operon expression, together with a nonlinear demand for end product, making its expression cyclic. The cell growth and dilution rates (related to the cell cycle and the liquid residence time in a (semi-)continuous bioreactor) strongly influence the TRP system stability, as proven in silico by Maria [33] and Maria et al. [34].

The adopted hybrid kinetic model is that of Maria [35] built up using the kinetic data of Chen [74] collected in an FBR operated under the nominal (nonoptimal) conditions of (Table 1), using the T5 strain of *E. coli*. This complex structured kinetic model (presented in Table 1, Table 2 and Table 4) is a deterministic one. The CCM-based model core is the glycolysis dynamic model of Maria [48] validated using literature data.

To keep the bilevel hybrid model of Maria [33,35] adapted here with a reasonable extension, as well as to facilitate estimation of its rate constants, this dynamic model accounts for only the key species included in four linked cell reaction modules [a–c, X] responsible for the TRP synthesis. Three modules concern the following cell processes [35]:

Module [a]—glycolysis with a modified GLC uptake system (due to the used modified *E. coli* T5 strain);

Module [b]—ATP recovery system;

Module [c]—TRP operon expression.

The fourth kinetic module concerns the biomass [X] growth dynamics in the FBR bulk. This last module is connected to the cell processes, by influencing the GLC dynamics in the bulk phase through the X growth rate (Table 2), which, in turn, influences the GLC import flux V1 into the cell (Table 3). The dynamic model is hybrid (bilevel) because it connects the macro state variables of the FBR (biomass X, GLC, TRP) with the cell nano-level key variables (GLC, F6P, FDP, PEP, PYR, and ATP; Table 2 and Table 3) of the glycolysis and those (TRP, OR, OT, and mRNA) of the TRP operon expression (Table 4). All four kinetic modules are linked to the macroscopic FBR dynamic model through the formulated mass balances (Table 1, Table 2 and Table 4).

The thus obtained hybrid extended kinetic model includes a large number (49) of rate constants. To facilitate the estimation rule and to avoid suboptimal estimates (i.e., rate constants, *locally* valid in the operating parametric space), only (27) independent rate constants were accounted for in the estimation step [35]. Moreover, a step-by-step estimation methodology was applied by Maria [35], by decomposing the estimation problem in successive subproblems of smaller dimensions, by taking advantage of the modular construction of the cell FBR hybrid model. The estimated rate constants by Maria [35] were validated by fitting the experimental key species kinetic curves of Chen [74] recorded in the FBR of (Table 1) over a long batch (63 h), using the novel *E. coli* T5 strain of Chen et al. [73], as well as by comparison with the literature data reviewed by Maria [34,36,37,48,49]. In short, the methodology used by Maria [35] to estimate the adopted bilevel modular dynamic model consisted of a sequence of *trial-and-error* steps, by adjusting the literature information (reaction rate expressions and constants characterizing the dynamics of cell metabolic species of interest) to fit the available experimental kinetic data recorded from the above-described FBR. The sequence of computational steps is summarized below.

#### 3.1.1. The Biomass [X] Growth

The cell culture in the bioreactor is considered to be homogeneous and introduced as a lump “X” in the FBR model (Table 2). A modified Contois model, modified by considering a power-law inhibition with the first-order growing biomass at the denominator [85], was proven to be the most adequate vs. the experimental data (Figure 8c). To overcome the absence, at this modeling stage, of the predicted values of [X] and [GLC] (coming from the FBR coupled with the glycolysis dynamic models), simulations of the biomass dynamics over the batch were performed using the experimentally recorded [X] and [GLC] species trajectories of Chen [74], interpolated with the cubic splines functions (INTERP1 facility of Matlab^TM^ package). The estimated kinetic model of the biomass is given in Table 2.

#### 3.1.2. The FBR Dynamic Model

The FBR ideal model of Maria [35] was adopted to describe the key species dynamics during the batch at a macroscopic level (in the liquid bulk phase). The bioreactor initial conditions and the time stepwise dynamics of the control variables (added GLC substrate solution concentration, and the feed flow rate F_L_) were further explored to obtain the desired optimum operation of the studied FBR.

The bioreactor ideal model main assumptions were as follows [2]: (i) isothermal, iso-pH, iso-DO operation; (ii) it is self-understood that nutrients, additives, antibiotics, and pH-control compounds are added initially and during FBR operation to ensure the optimal grow of the biomass, as indicated by Chen [74]; (iii) oxygenation with pure oxygen in excess over the batch to ensure an optimal biomass maintenance, and to contribute to the liquid homogeneity; (iv) perfectly mixed liquid phase (with no concentration gradients, see Table 1), of a volume increasing according to the liquid feed flow rate time-varying policy; (v) the limits of the liquid feed flow rate ($F_{L,j}$ in Table 2) are adjusted to not to exceed the bioreactor capacity (Max($V_{L}$*)* in Table 1); (vi) negligible mass resistance to the transport of oxygen and compounds into the liquid and biomass flocks (if any); (vii) GLC substrate is initially added in the bioreactor and during the batch according to an optimal feeding policy to be determined; (viii) the feed flow rate during the batch $F_{L,j}$ is varied according to an optimal feeding policy to be determined for every *“time-arc”* index J in Equation (5).

From a mathematical point of view, in a general form, the FBR dynamic hybrid model (Table 1, Table 2 and Table 4) translates to a set of 12 differential mass balances (ODE set) written as below for the key species of the FBR.:

Species in the bulk-phase:

(1) $$\frac{dc_{i}}{dt}=\frac{F_{L,j}}{V_{L}}\;\left(c_{inlet,i,j}\;-\;c_{i}\right)\;\pm \;r_{i}\left(c(t),c_{0},k\right)\;;\;c_{i,0}=c_{i}\left(t=0\right),$$

where index “*i*” denotes species present in the FBR bulk (GLC, TRP, PYR, X); index “*j*” denotes the FBR feeding time-arcs; *j* = 1, …, $N_{div}$.

Key species inside cells:

(2) $$\frac{dc_{i}}{dt}=\pm \;r_{i}\left(c(t),c_{0},k\right)\;-\;\mu \;c_{i}\;;\;c_{i,0}=c_{i}\left(t=0\right),$$

where index “*i*” denotes species inside cells, i.e., GLC, F6P, FDP, PEP, PYR, and ATP for glycolysis and OR, mRNA, E, and TRP for TRP operon expression.

Biomass in the bulk phase:

(3) $$\frac{dX}{dt}=r_{x}\left(c(t),c_{x,0},k\right)\;;\;c_{x,0}=c_{x}\left(t=0\right).$$

Liquid volume dynamics:

(4) $$\frac{dV_{L}}{dt}=F_{L,j}\;;\;V_{L,0}=V_{L}\left(t=0\right).$$

In Equation (1), $c_{inlet,i,j}$ refers to the concentration of the species index “*i*” in the feeding solution, constant over the time interval index “*j*” (*j* = 1, …,$N_{div}$). In the present case, only GLC is fed into the FBR during the batch. The reaction rate *r_i_* expressions together with the associated rate constants and other details are given in Table 1 and Table 4. In Equations (1)–(3), *c* is the vector of species concentrations, *c_o_* is the initial value of *c* (at time t = 0) given in (Table 1), and is the vector of the model rate constants. The reactor content dilution (determined by the increasing *V_L_* in Equation (4) is due to the continuously added *F_L_* term.

In Equation (1), GLC and *F_L_* are the control variables. The optimal $F_{L,j}$ to be determined is given for time stepwise values over *j* = 1, …, $N_{div}$ time-arcs. For instance, for the adopted $N_{div}$ = 5, *j* = 1, …, $N_{div}$ time-arc switching points given in Equation (5) are T1 = $\;t_{f}$/$N_{div}$ (12.5 h), T2 = 2 $\;t_{f}$/$N_{div}$ (25 h), T3 = 3 $\;t_{f}$/$N_{div}$ (37.5 h), T4 = 4$\;t_{f}$/$N_{div}$ (50 h), and $\;t_{f}$ = 63 h.

Feed flow-rate policy:

(5) $$F_{L,j}=\{\begin{array}{c} F_{L,0}\;\mathrm{if}\;0\;\leq t\;<\;T1\;\\ F_{L,1}\;\mathrm{if}\;T1\;\leq t\;<\;T2\\ F_{L,2}\;\mathrm{if}\;T2\;\leq t\;<\;T3\\ F_{L,3}\;\mathrm{if}\;T3\;\leq t\;<\;T4\\ F_{L,4}\;\mathrm{if}\;T4\;\leq t\;<\;t_{f} \end{array}.$$

Similarly, for the adopted $N_{div}$ = 5 equal time-arcs, the feeding policy for the GLC solution concentration is

(6) $$c_{glc,j}^{feed}=\{\begin{array}{c} c_{glc,0}^{feed}\;\mathrm{if}\;0\;\leq t\;<\;T1\;\\ c_{glc,1}^{feed}\;\mathrm{if}\;T1\;\leq t\;<\;T2\\ c_{glc,2}^{feed}\;\mathrm{if}\;T2\;\leq t\;<\;T3\\ c_{glc,3}^{feed}\;\mathrm{if}\;T3\;\leq t\;<\;T4\\ c_{glc,4}^{feed}\;\mathrm{if}\;T4\;\leq t\;<\;t_{f} \end{array}.$$

To not complicate the engineering calculus, the main assumption in Equations (5) and (6) is the following: on each time stepwise “arc”, index *j* = 1, …, $N_{div}$, the control variables $F_{L,j}$ and $c_{glc,j}^{feed}$ are kept constant. Of course, the values on each time-arc do not have to be necessarily equal to each other.

The “nominal” FBR nonoptimal operating conditions. Under the conditions of Chen [74], the control variables $F_{L,j}$ and $c_{glc,j}^{feed}$ are kept constant on each time-arc at the nonoptimal values given in (Table 1). Moreover, they are also the same, i.e., $F_{L,0}=F_{L,1}=F_{L,2}=F_{L,3}=F_{L,4}$, and $c_{glc,0}^{feed}=c_{glc,1}^{feed}=c_{glc,2}^{feed}=c_{glc,3}^{feed}=c_{glc,4}^{feed}$.

FBR optimal operating conditions. By contrast, under the optimal conditions studied in this paper, the suitable time stepwise values $F_{L,0}\;-\;F_{L,4}$ and those of $c_{glc,0}^{feed}\;-\;c_{glc,4}^{feed}$ are to be determined together (simultaneously) to reach the optimum of an objective function (maximum of TRP production here). Multi-objective FBR optimization is also possible (see [89,90]) but is beyond the scope of this paper.

#### 3.1.3. Module [a] Glycolysis and Module [b] ATP Recovery System

Once a dynamic model able to predict the biomass dynamics in the FBR was adequately fitted (Section 3.1.1), two other kinetic modules were considered for rate constant estimation, i.e., glycolysis *module [a]) and ATP recovery system (module [b]). Their reduced reaction pathways are given in Figure 3, while the reaction rate expressions are given in Table 2 and Table 3. The model of Maria [35] was adapted on the basis of the literature information [33,34,37,48,52], by performing only a few modifications in the reaction rate expressions to reflect the modified GLC import system of the modified *E. coli* T5 strain schematically represented in Figure 2B. The two cell modules [a] and [b] are interconnected by sharing the ATP species, while the module [a] and the [X] (kinetic) module are interconnected by sharing [X] and [GLC] species concentrations. Thus, the dynamics of species belonging to the three interconnected modules ([a], [b], and [X] kinetic model]) can be simulated concomitantly, according to the reduced reaction pathway of Figure 3. At this point, by adopting the rate constants from the literature for the coupled modules [a], [b], and [X], as a first guess (self-understood including the approximate PEP consumption), Maria [35] re-estimated the all rate constants of the ([a], [b], and [X]) kinetic models to fit the experimental kinetic data (i.e., the species dynamic trajectories recorded by Chen [74] in the FBR of Table 3) under the “nominal” operating conditions. The results are presented in Table 1 and Table 2. For supplementary details, the reader is referred to Maria [35].

In short, glycolysis module [a] is a determined sequence of 10 enzyme-catalyzed reactions (see the reduced pathways of Figure 2 and Figure 3 with only six lumped reactions) that convert glucose (GLC) into pyruvate (PYR). The free energy released by the subsequent TCA originating from PYR is used to form the high-energy molecules ATP and NADH that support the glycolysis and several enzymatic syntheses in the cell [91]. Adequate modeling of the glycolysis dynamics is important because the glycolytic intermediates provide entry/exit points to/from glycolysis. Thus, most of the monosaccharides, such as fructose or galactose, can be converted to one of these intermediates, further used in subsequent pathways. For example, PEP is the starting point for the synthesis of essential amino acids (AAs) such as tryptophan, cysteine, arginine, and serine [37,52,88,92].

Due to the tremendous importance of glycolysis in simulating the cell CCM, intense efforts have been made both in its experimental study and in modeling the dynamics of this process specifically in bacteria (short reviews [33,48,93]). The large number of glycolysis reduced or extended kinetic models proposed in the literature (review [48]) present a complexity ranging from 18–30 species, including 48–52 reactions, with a total of 24–300 or more rate constants. Most of these models are, however, too complex to be easily identified from (often) few available kinetic data and too complex to be further used for engineering calculations. Moreover, with a few exceptions, most of them cannot satisfactorily reproduce the occurrence of glycolytic oscillations on a mechanistic basis [33,36].

The adopted glycolysis kinetic model of Maria [33,48] even if of a reduced form, by accounting only for nine key species in lumped reactions including 17 easily identifiable rate constants belonging to V1–V6 metabolic fluxes (Figure 3, and Table 2 and Table 3) has been proven to adequately reproduce the cell glycolysis under steady-state, oscillatory, or transient conditions according to (i) the defined glucose concentration level/dynamics in the bioreactor bulk (liquid) phase, (ii) the total A(MDT)P cell energy resources, and (iii) the cell phenotype characteristics related to the activity of enzymes involved in the ATP utilization and recovery system (here denoted as module [b]). Detailed discussions about the operating conditions leading to glycolytic oscillations were extensively presented by Maria [33,36,37]. For this reason, the FBR and the glycolysis dynamic models have to be considered together (Table 2 and Table 3) when simulating the dynamics of the [GLC] in the FBR bulk phase and of the metabolites of interest (F6P, FDP, PEP, PYR, and ATP) into the cell. The adopted rate expressions for the glycolysis main fluxes V1–V6 presented in Table 2 and Table 3 are those of the basic model, except those of the GLC import system (V1), modified to match the T5 *E. coli* strain kinetic data [35]. It is worth mentioning that, even if not the case here, under certain conditions (i.e., external/environmental and internal/genomic factors), glycolysis and TRP synthesis can become oscillatory processes [34,36,37,48,49]. According to the experimental data, the produced TRP (module [c]) is excreted (Figure 3) through a process described by Chen [74]. The PYR key metabolite concentration in the cell is regulated through a complex mechanism [94,95], with the excess being excreted, as experimentally proven by Chen [74].

As revealed by the reactions in the pink square of Figure 3, the efficiency and the dynamics of the ATP recovery system are essential for the reaction rates of the whole CCM, as long as ATP plays a catalytic–chemical energy provider role. As underlined by Maria et al. [33,36,37], among the involved parameters, an essential factor is the k6 reaction rate (determined by the *ATPase* characteristics in Figure 3), included in the glycolysis model of Table 2 and Table 3. The involved enzymes characteristics are directly related to the cell phenotype (i.e., cell genomic) controlling the [AMDTP] total energy resources level. To not complicate the simulations, the [AMDTP] level was kept unchanged in the present analysis at an average value given in Table 1, as suggested by Chassagnole et al. [52]. The adopted kinetic model for the glycolysis (i.e., the V1–V6 reaction rates of Figure 3 and Table 2 and Table 3) and the equilibrium relationships for the ATP–ADP–AMP system given in Table 2 and Table 3 were imported from the literature [33,35,48]. This kinetic model was proven by Maria [35], according to experimental checks to fairly represent the dynamics and the thermodynamics of the internal modules [a,b] in the modified *E. coli* T5 strain.

#### 3.1.4. Module [c] TRP Synthesis

The adopted in silico evaluation of the TRP synthesis of Maria [35] is based on a simplified pathway displayed in Figure 3, derived from various studies reviewed by Maria et al. [37]. Modeling the TRP synthesis using a deterministic (mechanism-based) approach is difficult because this cellular process is known as being, under certain conditions, a QSS or an oscillatory one [33,81,83]. However, to avoid extended models, difficult to be estimated and used, most of the reduced dynamic models from the literature do not distinguish the process components from the regulatory components, and lumped reactions/species are considered instead, with the regulatory performance being included via adjustable model parameters and terms. Kinetic models trying to reproduce the TRP operon expression self-regulation [82,83] are too extended for our engineering evaluation purposes. Due to the process complexity, some modeling approaches [63] instead focused on determining correlations among flux distribution, flux control, and the optimized enzyme activity distribution, by employing a reduced kinetic model, not able to simulate most CCM key modules.

For such reasons, in the present analysis, simulations of the TRP synthesis were performed using the reduced CCM-based kinetic model of Maria et al. [33,37].

The adopted dynamic model of Maria [35] for the TRP synthesis (TRP operon expression) is given in Table 4. This kinetic model is a modification of those proposed by Bhartiya et al. [81]. The operon expression regulation terms (C1,C2) were kept unchanged. Only the TRP mass balance was changed according to the below reasons. The rate constants of the considered OR, mRNA, TRP, and E key species mass balances were re-estimated using the experimental data of Chen [74] given in Figure 4, Figure 5, Figure 6, Figure 7 and Figure 8. The TRP mass balance of the Bhartiya et al. [81] model was modified and re-estimated step by step as follows:

i. An explicit connection of the TRP module to the glycolysis module [a] pathway was introduced through the PEP precursor sharing node (in Figure 3). Consequently, PEP is included as a substrate in the TRP mass balance (dc_TRP_/dt in Table 4), while the PEP consumption term is also considered in the PEP balance of the glycolysis model according to the recommended fluxes ratios of Stephanopoulos and Simpson [88], as a first guess (Table 2). Analysis of this model suggests that intensifying TRP synthesis clearly depends on the glycolysis intensity (average levels of glycolytic species) and its dynamics (QSS or oscillatory). In fact, as remarked by Li et al. [78] and by Chen and Zeng [76], the PEP precursor is the limiting factor for TRP synthesis. This is why intense efforts have been made to increase its production through glycolysis intensification [33,34]. This can be realized by optimizing the FBR operating policy (as in this paper) and/or by using (also in this paper) the modified *E. coli* T5 strain culture of Chen et al. [73] and Chen [74].
ii. The TRP synthesis model of Bhartiya et al. [81] (Table 4) includes two terms for the product inhibition, i.e., the C3 term (of allosteric-type) plus a Michaelis–Menten term. Our tests proved that these terms do not adequately fit the TRP experimental kinetic data of Figure 4. Accordingly, the product inhibition term in the TRP balance of Table 4 was replaced by the more adequate Contois-type model, considering a power-law inhibition of the first-order growing TRP at the denominator. Eventually, the rate constants of the TRP [c] kinetic module, the PEP consumption stoichiometry, and the rate constants of the other modules ([a], [b], and [X]) were re-estimated (refined) simultaneously using the whole (complete) hybrid FBR model, as well as all available experimental kinetic trajectories of the key-species offered by Chen [74] (Table 1, and Figure 4, Figure 5, Figure 6, Figure 7 and Figure 8). The initial guesses of the rate constants of the TRP module [c] were adopted from the literature.
iii. The required PEP and GLC dynamic trajectories during estimation were transferred among the modules [a], [b], [c], [X] of the FBR kinetic model, all being available at this point.
iv. In contrast to the literature, in the TRP balance of Table 4, an activation inhibition term was considered by bringing together the substrate (PEP) and the first key enzyme (anthranilate synthase, E) that trigger TRP synthesis [35]. Such an approach was proven to better fit the experimental data of Figure 4, i.e., $\;c_{trp}(t_{u})$, *u* = 1, …, *n* (where *n* = 17 denotes the number of experimental points) and to confer more flexibility to the derived model. The estimated negative *g* constant, of a small negative value, reflects the slight inhibition of TRP synthesis with the substrate PEP, as also suggested in the literature [35].

### 3.2. Rate Constant Estimation by Maria (2021)

In total, the developed hybrid structured kinetic model includes 49 rate constants to be estimated from the experimental kinetic curves of four observed species (GLC, TRP, PYR, and X), with each species time trajectory including 17 uniformly distributed recorded points (Figure 4, Figure 5, Figure 6, Figure 7 and Figure 8). This estimation problem is equivalent to a nonlinear programming one (NLP) of high difficulty [41] due to its dimension, the high nonlinearity of the model, and its associated constraints.

To avoid unfeasible local estimates of the NLP problem, Maria [35] used a sequential approach. A rough estimate of the kinetic module [a] + [b] + [c] + [X] (Table 1, Table 2 and Table 4) rate constants was generated using a step-by-step (module-after-module) approach, also accounting for the shared species (PEP for [a] + [c]; X and GLC for [a] + [b] + [X]). If missing during simulations, the experimental TRP, GLC, or X time trajectories were taken instead (interpolated with the cubic splines INTERP1 facility of Matlab™ package [35]).

Finally, the thus obtained rate constants were refined by means of a standard weighted least square criterion [41] considering the whole FBR hybrid model, including all four interconnected modules [a], [b], [c], [X]. To reduce the problem size, only 27 independent model rate constants were accounted during estimation (from the total of 49 rate constants). A number of rate constants were adopted from the literature [34,37]. Eventually, all rate constants were refined by Maria [35], as presented in Table 1, Table 2 and Table 4. The thus identified FBR hybrid structured dynamic model fit the experimental data very well, as indicated by Figure 4, Figure 5, Figure 6, Figure 7 and Figure 8.

The multimodal NLP estimation problem solved by Maria [35] is a difficult one, being highly nonlinear, including nonlinear constraints defining a nonconvex domain. For such large nonconvex estimation problems, the usual optimization routines usually encounter difficulties in reaching the feasible global solution with an acceptable reliability. This is why a very effective NLP solver was used instead, i.e., the adaptive random search MMA of Maria [96] implemented on the Matlab^TM^ numerical calculus platform. The NLP solution was checked using several (randomly generated) *initial guesses* for the rate constants. A stiff integrator (ODE15S routine of Matlab^TM^ package) was used to solve the ODE dynamic model with a high accuracy.

A comparison of the model-estimated rate constants for the modified T5 *E. coli* strain using the FBR experimental data of Chen [74] with those of the same model but estimated from experiments using the wild *E. coli* strain was presented by Maria [35]. As expected, most of the estimated rate constants presented similar values for some reaction steps. However, due to the mentioned modifications of the used *E. coli* T5 strain in the present kinetic model, important differences were reported for (i) the rate expression and parameters of the GLC import system (V1 in Table 2 and Table 3, and Section 3.1.3), (ii) the biomass growing dynamics (Table 2), and (iii) the TRP synthesis module [c], in terms of both parameters and rate expressions (Table 3). As another observation, for the nominal (nonoptimal) FBR experimental conditions of Table 1 used by Chen [74], the species dynamics belonging to inside the cell and to the external liquid phase tend to reach a quasi-steady state (QSS) that corresponds to a balanced cell growth (homeostasis) in the bioreactor [35].

### 3.3. Ways to Intensify the TRP Production in the FBR

As revealed by the concerned literature [34,35,36,37,74], intensifying the TRP synthesis strongly depends on (a) the glycolysis intensity (GLC uptake flux, and average levels of glycolytic species of module [a]), transmitted to the TRP synthesis module [c] via the shared PEP intermediate, and (b) on the glycolysis dynamics (QSS, or oscillatory behavior) [33,34,37]. More specifically, as pointed out in the literature by Maria et al. [36] and Maria [33], the glycolysis intensity is controlled by both cell internal and external factors, as follows:

i. The GLC import system efficiency (V1 in Figure 3) is regulated and triggered by the external concentration of glucose and by the subsequent PEP and PYR synthesis (Table 2 and Table 3). The regular GLC uptake system, i.e., the PTS translocation system in the *wild* strain (of a complex reaction rate expression discussed by [35,48,52]) was replaced in the present studied *E. coli* T5 strain, as mentioned in Section 2, with a more efficient one (Figure 2B) able to accelerate the GLC uptake flux into the cell at least twofold [74]. Such a modified GLC import was modeled by simple Michaelis–Menten kinetics in the model of Table 3 by accounting for the well-known GLC substrate inhibition.
ii. The quick import of GLC and its conversion to the precursor PEP require important amounts of regenerable ATP and a rapid enough ATP-to-ADP conversion rate, as well as its quick regeneration. The re-estimated rate constants of the kinetic module [b] (pink rectangle in Figure 3, and Section 3.1.3), concomitantly with those of the kinetic module [a] from the experimental data coming from the FBR operated with modified *E. coli* cells implicitly ensure the requirement that the A(MDT)P energy system is able to support the cell glycolysis (see V2, V4, and V6 expressions in Table 3 and the ATP mass balance in Table 2). On the other hand, limited A(MDT)P energy resources which exist in the cell slow down the GLC import if the ATP use/regeneration is not working fast enough [97]. Such an A(MDT)P resource is linked to the microorganism phenotype. Here, the total A(MDT)P was adopted (Table 1 and Table 3) at the average level recommended by Chassagnole et al. [52].
iii. Additionally, due to the enzyme *ATPase* and *AKase* characteristics related to the bacteria genome and cell phenotype (Figure 3), a limited ATP conversion rate can sustain the glycolytic reactions, while the ATP recovery rate is limited by the enzymes participating in the A(MDT)P interconversion reactions (i.e., the K and k6 rate constants in the kinetic model of Table 3). This is why the k6 rate constant was re-estimated here to fit the experimental data, as suggested by Maria et al. [36,49].
iv. At the same time, as glycolysis is a systemic process, with a complex regulatory structure, its dynamics (oscillatory, transient, or QSS) is also related to the rate constants of all involved reactions. Consequently, all these rate constants were considered in the final estimation step of the whole FBR hybrid kinetic model. Similarly, Silva and Yunes [98] found that glycolysis (QSS or oscillatory) is only possible if the external concentration of GLC and the maximum reaction rates controlled by the enzymes *PFKase* and *GKase* (which control the V1 and V2 reactions of Figure 3) are within specific intervals. Due to the same reason, the rate constants related to the GLC uptake system in the modified *E. coli* cell (modified V1 flux in Table 3) were re-estimated to match the experimental kinetic data.
v. As a corollary of the issue (iv), Maria [33,34,36,37] determined the operating conditions leading to glycolytic oscillations or QSS by varying the external factor [GLC]ext and some internal factors such as the total [AMDTP] level and the k6 rate constant of Table 3. Such an investigation was not necessary here, because no oscillatory process was identified in the present operating case.
vi. Simulations by Maria [33,35] revealed that the TRP synthesis efficiency is also strongly influenced by external factors, related to the FBR operating regime, namely, (a) the cell dilution (taken into account as “μ” in the approached hybrid kinetic model of Table 2, (b) the GLC concentration in the external (bulk) phase ($c_{glc}^{ext}$ in Table 2), and (c) the optimal operating policy for the control variables. In this paper, such an operating policy will correspond to the time stepwise variation of the feed flow-rate ($F_{L,j}$ in Equation (5)) and of the GLC feeding concentration ($c_{glc,j}^{feed}$ in Equation (6)).

## 4. Fed-Batch Bioreactor Optimization Problem

### 4.1. Preliminary Considerations

To support further engineering calculations, a reasonable extended hybrid modular approach was adapted from literature [35], by expressing the macroscopic main state variable species dynamics (i.e., biomass X, GLC, and TRP) governing the FBR performance, as a function of intracellular species dynamics related to the cell CCM metabolic fluxes responsible for the TRP synthesis. This inner cell environment link is realized by means of model key species (GLC, X, PEP, ATP) (Section 3.1). The main modification in this paper of this adopted hybrid dynamic model refers to the introduction of a variable FBR feeding both in the feed flow rate F_L_ (Equation (5) and Table 2) and in the GLC feeding solution concentration (Equation (6) and Table 2).

The reasonable compromise between the hybrid model details (number of intracellular species and reaction pathways accounted for) and its predictive value was realized by using only the cell key-modules [a]–[c] of interest (Figure 3, in a lumped form, Section 3.1.3 and Section 3.1.4) linked to bulk phase species (X, GLC) (Section 3.1.2 and Section 3.1.3). The fair adequacy of the resulted dynamic model (Table 1, Table 2 and Table 4) vs. the experimental data was proven by Maria [35]. Consequently, this hybrid model becomes suitable for further engineering evaluations of the reactor and process efficiency, as is the case here.

The optimal FBR operation derived in this paper is more complex than the simple nonoptimal (“nominal”) operation of Chen [74] (Table 1). Mainly, the feed flow rate and GLC concentration in the feeding solution are no longer kept constant. In contrast, (i) the batch time is divided in *N_div_* (equal “time-arcs”) of equal lengths, and (ii) the control variables are kept constant only over every “time-arc” at optimal values for each time-arc determined from solving an optimization problem (i.e., maximization of the TRP production in this case). The time intervals of equal lengths Δ*t* = *t_f_*/*N_div_* are obtained by dividing the batch time *t_f_* into *N_div_* parts *t*_*j*−1_ ≤ *t* ≤ *t_j_*, where *t_j_* = *j*Δ*t* are switching points (where the reactor input is continuous and differentiable). Time intervals for the present case study with an adopted *N_div_* = 5 are shown in the “liquid volume dynamics” row of Table 2 and its footnote (a).

### 4.2. Formulation of the Optimization Problem

#### 4.2.1. Selection of the FBR Control Variables

By analyzing the FBR hybrid model of Table 2, completed by Table 3; Table 4, the natural option is to choose as control variables those with a high influence on the biological process, which are easily to handle. In the present case, according to the discussion of Section 3.3, two control variables were chosen related to the bioreactor feeding:

(a) The substrate $c_{glc,j}^{feed}$ (j = 1, …, $N_{div}$) whose concentration plays a major role in the cell glycolysis and TRP production;
(b) The liquid feed flow rate $F_{L,j}$ (*j* = 1, …, $N_{div}$), with a GLC solution directly linked to the GLC feeding, responsible for the reactor content dilution.

In the present optimization strategy, each control variable is kept constant over each time-arc (index “*j*”). Of course, they are not necessarily equal between different time-arcs. For $N_{div}$ = 5, in total there are 5 × 2 = 10 unknowns in Equation (7) to be determined by optimization, under certain constraints (Table 2):

(7) $$F_{L,j};\;c_{glc,j}^{feed},\;(j=1,\;\ldots ,\;N_{div}).$$

The FBR initial state is given in Table 1 for both inside cell and bulk-phase species. Those of the control and bulk phase variables, i.e., the initial liquid flow rate and the substrate initial concentration (as shown in Table 2, and Equations (5) and (6)) are included as unknown variables in the FBR optimization, i.e.,

*F*_*L*,0_ = *F_L_* (*t* = 0), (8)

in Equation (5), and

(9) $${\left[\mathrm{GLC}\right]}_{0}=c_{glc}^{ext}(t=0)=c_{glc,0}^{feed},$$

in Equation (6).

#### 4.2.2. Objective Function (Ω) Choice

By considering the mentioned control variables Equation (7), the FBR optimization consists of determining its optimal initial load simultaneously with its feeding policy for every time interval during the batch, eventually leading to maximization of the [TRP] production during the batch.

The control variables values of Equations (7)–(9) to reach Max Ω were identified, where

Ω = Max [TRP(*t*)], with (*t*) ∈ [0, *t_f_*]. (10)

The [TRP](*t*) dynamics in Equation (10) was evaluated in silico by solving the ODE dynamic model of the FBR (Equations (1)–(6)) over the whole batch time (*t*) ∈ [0, *t_f_* ].

#### 4.2.3. Optimization Problem Constraints

The optimization problem in Equation (10) was subjected to the following multiple constraints:

(a) The FBR model in Equations (1)–(6) including the bioprocess kinetic model (Table 1, Table 2 and Table 4);
(b) The FBR initial condition from Table 1, except $F_{L,0}$ and $c_{glc,0}^{feed}$ which were determined from solving the optimization problem (the initial guess was taken from Table 1);
(c) To limit the excessive consumption of substrate and to prevent the hydrodynamic stress due to the limited reactor volume, feasible searching ranges were imposed on the control/decision variables, i.e.,
  [GLC]_inlet,min_ = 1000 (mM) ≤ [GLC]_inlet,j_ ≤ [GLC]_inlet,max_ = 4500 (mM), *F*_*L*,__min_ = 0.01 (L/h) ≤ *F_L,__j_*; *F*_*L*,0_ ≤ *F*_*L*,__max_ = 0.04(L/h); (11)
(d) Physical meaning of searching variables:
  (12) $$F_{L,j}\;>\;0;\;c_{glc,j}^{feed}\;\geq \;0\;(j=1,\;\ldots ,\;N_{div});$$
(e) Physical meaning of state variables:
  (13) $$c_{i}\;(t)\;\geq \;0\;(i=1,\;\ldots ,\;\mathrm{number}\;\mathrm{of}\;\mathrm{species}\;\mathrm{in}\;\mathrm{the}\;\mathrm{model});$$
(f) Limit the maximum cell resources in AMDTP:
  [ATP] (*t*) < Total [AMDTP], with [ATP] (*t*) obtained from solving the FBR model in Equations (1)–(6). (14)

As an observation, the imposed ranges for the control variables were related to not only the implementation facilities, but also economic reasons, achieving minimum substrate consumption, reduced dilution of the reactor content, and an effective bioreactor control.

#### 4.2.4. $N_{div}$ and Operating Alternatives Choice

The adopted FBR operating policy alternative of Section 4.2.1 is one of the simplest variable operating modes. It implies a time stepwise variable feeding of the bioreactor, over an adopted ($N_{div}$ = 5 here) equal time-arc that covers the whole batch time. Each time-arc “*J*” (*j* = 1, …, $N_{div}$) is characterized by optimal levels of the feed flow rate $F_{L,j}$ and of the GLC concentration $c_{glc,j}^{feed}$ (see Equations (7)–(9)).

This type of FBR operation, despite its simplicity and easy implementation, still includes enough degrees of freedom to offer a wide range of operating facilities that, in principle, might be investigated, for instance (see also the discussion of Maria [3]), (a) by choosing unequal time-arcs, of lengths to be determined by the optimization rule, (b) by considering the whole batch time as an optimization variable, (c), by increasing the number of equal time-arcs ($N_{div}$) to obtain a more *refined* and versatile FBR operating policy, but keeping the same nonuniform feeding policy (of the two control variables here), (d) by considering the search min/max limits of the control variables as unknown (to be determined), or (e) by feeding the bioreactor with a variable feed flow rate, but with a GLC solution of an uniform concentration over a small/large number ($N_{div}$) of time-arcs. All alternatives (a–e) are not approached here for the reasons discussed below.

Alternatives (a–c) are not good options, because, as $N_{div}$ increases, the necessary computational effort grows significantly (due to a considerable increase in the number of searching variables), thus hindering the quick (real-time) implementation of the derived FBR operating policy. Additionally, multiple optimal operating policies can exist for the resulting overparameterized constrained optimization problem of a high nonlinearity, thus increasing the difficulty in quickly locating a feasible globally optimal solution of the FBR optimization problem.

Additionally, as the $N_{div}$ increases, the operating policy is more difficult to implement, since the optimal feeding policy requires a larger number of stocks with feeding substrate solutions of different concentrations, separately prepared to be fed for every time-arc of the FBR operation (an overly expensive alternative). Moreover, the NLP optimization problem is more difficult to solve because of the multimodal objective function, leading to multiple solutions difficult to discriminate and evaluate. This is the case, for instance, of an obtained infeasible optimal policy requiring a very high [X], difficult to be ensured due to limitations in keeping the necessary levels of the related running parameters of the bioreactor (i.e., dissolved oxygen, nutrients, pH-control substances, antibodies, etc.). Furthermore, FBR operation using a larger number of small time-arcs $N_{div}$ can raise special operating problems when including PAT (process analytical technology) tools [99].

A brief survey of the FBR optimization literature [100,101,102] reveals that a relatively small number $N_{div}$ < 10 is commonly used for such an FBR due to the abovementioned reasons. In fact, the present numerical analysis does not intend to exhaust all the possibilities of the approached FBR optimization. Thus, an extended analysis of the operating alternatives (a–d) of the FBR operation or the influence of the parametric uncertainty deserves a separate investigation, beyond the scope of this paper. To not complicate the computational analysis, only $N_{div}$ = 5 equal time-arcs are tested here, with equal time-arc lengths of $t_{f}$/$N_{div}$ = 63/5 h.

The alternative (d) is unlikely because it might indicate unrealistic results, as explained in point (c) of Section 4.2.3. In our numerical analysis, carefully documented upper bounds of control variables were tested to ensure the practical implementation of the optimal operating policy.

Alternative (e) is also not feasible, even if a larger $N_{div}$ is used. That is because it is well known that the variability of the FBR feeding over the batch time-arcs is the main degree of freedom used to obtain FBR optimal operating policies of superior quality [3,6,89,101]. By neglecting the variable feed flow rate and substrate concentration, suboptimal FBR operating policies will be obtained of low performance.

#### 4.2.5. The Used Numerical Solvers

The prediction of the species concentration time evolution inside the cell and in the bulk phase was obtained by solving the FBR dynamic model in Equations (1)–(6) with the initial condition of *C*_*j*,0_ = *C_j_* (*t* = 0) of Table 1 for the inside cell species, except the bulk [GLC]_0_ to be determined from the FBR optimization, as indicated by Equations (7) and (9). The imposed batch time *t_f_* and the optimal medium conditions are those of Table 1. The dynamic model solution was obtained with a high precision, using the high-order stiff integrator (“ode15s”) of the MATLAB™ numerical calculus platform, with suitable quadrature parameters to keep the integration error very low.

Because the FBR hybrid model structure in Equations (1)–(6), its reaction rate terms (Table 1, Table 2 and Table 4), and the problem constraints from Equations (11)–(14) (Section 4.2.3) are all highly nonlinear, the formulated problem in Equations (7)–(10) translates into a nonlinear optimization problem (NLP) with a multimodal objective function and a nonconvex searching domain. To obtain the global feasible solution with enough precision, the multimodal optimization solver MMA of Maria [41,96,103] was used, proven in previous studies to be more effective compared to the common (commercial) algorithms. The computational time was reasonably short (minutes) using a common PC, thus offering a quick implementation of the obtained FBR optimal operating policy [96,103].

#### 4.2.6. The Problem Solution Particularities

The obtained optimal operating policy of the FBR, for the optimization problem formulated in the Section 4.2.2, with the control variables of Section 4.2.1, the constraints of Section 4.2.3, and adopted $N_{div}$ in Section 4.2.4, is given in Figure 7 for the feeding policy of the GLC concentration $c_{glc,j}^{feed}$ (j = 1, …, 5) and in Figure 8a for the feed flow rate $F_{L,j}$ (j = 1, …, 5). It is to be observed that, due to the above formulated engineering problem, the FBR optimal operating policy is given for every time interval (of equal length) uniformly distributed throughout the batch time.

Such an optimal time stepwise variable feeding of the bioreactor presents advantages and inherent disadvantages. The advantages are related to the higher flexibility of the FBR operation, leading to a higher productivity in TRP, as proven in Section 5. Furthermore, the imposed limits of the control variables prevent excessive substrate consumption or an excessive reactor content dilution.

As a disadvantage, FBRs with such time-variable control are more difficult to operate than simple BRs, as long as the time stepwise optimal feeding policy requires different stocks of feeding substrate solutions of different concentrations to be used over the batch. This is the price paid for achieving the best performance of an FBR. This need to previously prepare different substrate stocks to be fed for every “time-arc” (i.e., a batch-time division in which the feeding is constant) is offset by the net higher productivity of FBR compared to that of BR as discussed below and pointed out in the literature [6,89,90,101,104]. In fact, the best operating alternative (FBR vs. BR) is related to many other economic factors (operating policy implementation costs, product cost compared to production costs, product price fluctuation, etc.), not discussed here.

## 5. Optimization Results and Discussion

The obtained optimization problem solution (of the type discussed in Section 4.2.6) is given in Figure 7 (top, curve 2) for the GLC feeding concentrations and in Figure 8a (curve 2) for the feed flow rate. The optimally operated FBR displays the bulk [TRP] dynamics of Figure 4 (curve 2). The corresponding dynamics of cell glycolytic species during the batch is presented in Figure 5, while that belonging to the TRP operon expression is presented in Figure 6. The dynamics of species present in the reactor liquid phase are presented in Figure 7 for GLC and in Figure 8c for the biomass (X). In these figures, the species dynamics plotted for the optimal FBR operation (black curve 2, i.e., the model predictions) are compared to those corresponding to the nominal, nonoptimal FBR operation (blue curve 1 of Maria [46]) and with the experimental results (blue points) of Chen [71]. Both operating policies (optimal 1 and nonoptimal 2) are obtained using the same modified *E. coli* T5 strain of Chen [70,71].

By analyzing the resulting FBR optimal operating policy (plot no. 2 in Figure 4, Figure 5, Figure 6, Figure 7 and Figure 8) compared to the suboptimal (nominal) operation of Chen [71] (plot no. 1 in Figure 4, Figure 5, Figure 6, Figure 7 and Figure 8), several observations can be derived, as follows:

By using the same FBR operated under nominal (nonoptimal) conditions of Table 1, the modified *E. coli* T5 strain reported a higher GLC uptake rate and a much higher TRP production compared to the “wild” strain, as revealed by the analysis given in Table 5.

The efficiency of the optimally operated FBR (this paper) in TRP production is significantly higher (ca. 20%) compared to the same FBR but suboptimally (nominal) operated (Table 5), even if the same modified *E. coli* T5 strain is employed in both cases. The same conclusion also results by comparing the TRP final concentrations in the FBR bulk given in Figure 4 for the two operating policies.

The optimal FBR operation reported a similar dilution of the reactor content, as revealed by (Figure 8b).

The substrate (GLC) consumption in (Table 5) was computed using the following relationship:

(15) $$m_{GLC}=\sum_{j=1}^{N_{\mathrm{div}}}c_{glc,j}^{feed}\;F_{L,j}\;\Delta t_{j}\;;\;\Delta t_{j}=t_{f}{/N}_{\mathrm{div}}\;.$$

As expected, a higher TRP productivity requires a higher GLC consumption, as is the case when using a modified *E. coli* T5 strain instead of the “wild” type. As revealed by (Table 5), the GLC consumption is influenced by the FBR operating mode, even if the same cell strain is used. As indicated by our present analysis given in Table 5, the GLC overall consumption for the optimal (variable feeding) FBR operation is roughly similar to that of a nonoptimal (uniform feeding) FBR operation. Not surprisingly, the optimal operating mode requires a slightly lower GLC consumption (ca. 6%) because of its better use during the batch.

The comparative analysis of the glycolytic species dynamics in Figure 5 reveals close trajectories (even quasi-identical for F6P, FDP species), without any accumulation tendency, for both nominal (nonoptimal, curve 1) and optimal (curve 2) FBR operation. By contrast, the intermediate PEP intermediate species is formed in high amounts but then quickly consumed in the subsequent TRP synthesis, thus tending to reach a QSS. Such a more intensive GLC import for the optimal FBR operation (curve 2) and its successive transformation over the glycolysis pathway and TRP-operon expression are reflected by a higher ATP consumption compared to the nonoptimal FBR operation. The PYR metabolite is consumed in the TCA cycle and excreted in the bulk phase (fairly predicted by our kinetic model matching the experimental data).

The comparative analysis of the TRP operon expression species dynamics in Figure 6 reveals very close trajectories, except for the excreted TRP, for both nominal (nonoptimal, curve 1) and optimal (curve 2) FBR operation. Such a result can be explained by the operon expression mechanism, involving a tight control via its inhibition terms presented in Table 4.

The comparative plots of the GLC concentration dynamics in the FBR bulk phase are presented in Figure 7. They indicate similar decreasing trajectories for both investigated FBR operating alternatives, i.e., (i) nominal (nonoptimal, curve 1) and optimal (curve 2). Such a result can be explained by the same GLC uptake mechanism of the modified *E. coli* T5 strain. In the optimal case (curve 2), the GLC consumption is higher, due to a higher TRP productivity. The curve 2 unevenness is linked to the variable feeding with GLC of the optimally operated FBR (see the feeding plots in the top part of (Figure 7)).

The comparative plots of the biomass dynamics in the FBR bulk phase are presented in Figure 8c. They reveal similar increasing trajectories for both investigated FBR operating alternatives, i.e., (i) nominal (nonoptimal, curve 1) and (ii) optimal (curve 2). In the optimal operation case, the biomass growth is more intense, due to a significantly higher GLC uptake and a better GLC use during the batch, thus offering more favorable conditions for the biomass growth.

The TRP concentration dynamics in the bulk phase is plotted in Figure 4 for both investigated FBR operating alternatives, i.e., (i) nominal nonoptimal operation of Table 1 (curve 1) and the experimental data (●, blue) of Chen [71], and (ii) optimal operation (curve 2). The TRP higher final concentration leads to a higher productivity for the optimally operated FBR (see observation 2 above). Such a result proves that the optimal time stepwise FBR feeding (i.e., the GLC feeding curve 2 in Figure 7) and the feed flow rate policy of Figure 8a) is superior to the nonoptimal uniform feeding, leading to a better GLC use, even if the overall GLC consumption (see observation 4 above) is similar for both nominal and optimal FBR operation. The better GLC use for the optimal FBR operation is also proven by the less produced secondary metabolite PYR in Figure 5 (curve 2) and by the smaller QSS concentration of the PEP intermediate (Figure 5, curve 2), quickly transformed into the final product TRP.

## 6. Conclusions

The extended bilevel (hybrid) kinetic model adopted in this paper was proven by Maria [46] to adequately represent the dynamics of an experimentally studied FBR under a nominal (uniform feeding) operating policy, for both macroscopic state variables and for the cell key species of the CCM reaction modules related to the TRP production in the FBR, i.e., [a] glycolysis, [b] ATP recovery system, [c] TRP operon expression, and biomass [X] growth. The hybrid structured model, linking the macro state variables to the nano cell-scale variables, was validated using the recorded data from the lab-scale FBR over a long batch time (63 h).

By adopting this adequate kinetic model, the paper exemplifies how the use of reduced CCM-based hybrid kinetic models, of modular construction, including the inter-connected complex metabolic pathways of interest, is a continuously challenging subject when developing structured cell simulators for various engineering applications, such as (a) metabolic flux analysis under variable operating conditions, (b) target metabolite synthesis optimization by optimizing the bioreactor operation, and/or by modifying the cell strain, (c) in silico reprogramming of the cell metabolism to design GMOs (not approached here), (d) a quick analysis of the cell metabolism, leading to an evaluation of substrate utilization, oscillation occurrence, and reactor QSS conditions or structured interpretations of the metabolic changes in modified cells or in direct connection to the bioreactor operation mode, (e) bioreactor/bioprocess optimization (the present study), (f) to derive simple lumped models, locally valid (in the operating parameters domain), and (g) to allow more robust extrapolations of the bioprocess behavior (not tested here).

The engineering evaluations developed in this paper can be further extended, for instance, by deriving a multi-objective optimization of the FBR operating policy, by accounting for not only maximization of the product (TRP), but also minimization of the substrate consumption and of the batch time. The engineering evaluations using such extended bilevel hybrid models present a higher prediction accuracy compared to the simple global (empirical) dynamic models.

Simulations and the experimental checks proved the advantage of using the modified *E. coli* T5 strain culture to improve the TRP production. The obtained results also proved that, in addition to the cell phenotype characteristics (linked to GLC uptake and glycolysis), the FBR operation mode is the major factor determining the TRP synthesis efficiency.

## Figures and Tables

**Table 4:** The mass balances in the kinetic module [c]. Species mass balances in the TRP operon expression kinetic model of Bhartiya et al. [81] were modified by Maria et al. [37] to better fit the experimental data, as follows: (i) PEP (from glycolysis) is the substrate of TRP synthesis and the node coupling this synthesis with the glycolysis [a] module; (ii) a novel model for the TRP synthesis inhibition was proposed and identified from experiments. The model rate constants were estimated by Maria [35] to fit the experimental data of Chen [74] (Figure 4, Figure 5, Figure 6, Figure 7 and Figure 8) collected in the FBR using the modified *E. coli* T5 strain, under “nominal” operating conditions (Table 1). Species notations (TRP, OR, OT, mRNA, and E) are given in the abbreviation list. QSS = quasi-steady state.

Rate Expression	Kinetic Model Parameters (Units in mM, μM, min)
$\frac{dc_{OR}}{dt}=k_{1}c_{OT}C_{1}(c_{trp})-k_{d1}\;c_{OR}-\mu \;c_{OR}$ $\frac{dc_{MRNA}}{dt}=k_{2}c_{OR}C_{2}(c_{trp})-k_{d2}\;c_{MRNA}-\mu \;c_{MRNA}$ $\frac{dc_{E}}{dt}=k_{3}\;c_{MRNA}-\mu \;c_{E}$ $C_{1}(c_{trp})=\frac{K_{i,1}^{n_{H}}}{K_{i,1}^{n_{H}}+\;c_{trp}^{n_{H}}}$; $C_{2}(c_{trp})=\frac{K_{i,2}^{1.72}}{K_{i,2}^{1.72}+\;c_{trp}^{1.72}}$	$k_{1}$ = 59.062, 1/min·mM $k_{d1}$ = 0.5443, 1/min $k_{2}$ = 17.796, 1/min $k_{d2}$ = 14.094, 1/min $k_{3}$ = 1.157, 1/min $K_{i,1}^{}$ = 3.53, μM $n_{H}$ = 1.92 $K_{i,2}^{}$ = 0.04, μM (see footnote (d))
$\frac{dc_{trp}}{dt}=\;{\left(c_{pep}c_{E}\right)}^{g}\frac{\mu_{T}\;c_{trp}}{{\left(a_{T}\;exp\left(b_{T}\;t\right)\right)}^{N_{T}}}\;-\mu \;c_{trp}$ (see footnotes (a)–(d))	*g* = −0.32 $\mu_{T}\;$= 0.36365, 1/min $a_{T}$ = 3.9923 $b_{T}$ = 0.017153, 1/min $N_{T}$ = 0.071515

(a) The adopted modification for the TRP synthesis inhibition replaces the C3 variable of the Bhartiya et al. [81] model (not displayed here, see [35]) with a modified Contois model, including a power-law inhibition with TRP growth at the denominator. (b) The nitrogen source in the TRP synthesis is considered in excess and included in the model constants. (c) To be connected to the glycolysis kinetic model, the PEP species dynamics, generated by the glycolysis model, was explicitly included in the TRP synthesis rate as a substrate [35]. (d) The initial concentrations of the TRP operon species (OR, mRNA, E, and TRP) are given in Table 1.

**Table 5:** Efficiency of the modified *E. coli* T5 strain for GLC uptake and for the TRP production in the tested FBR of (Table 3).

*E. coli* Strain	V1 Flux (in the Initial FBR Conditions) (mM/min)	Total GLC Consumption over the Batch Time (g)	TRP-Production of FBR (mM/min)
Maria et al. [34] (wild strain)	1.2485 × 10^2^	360	0.001–0.04 (nonoptimized FBR)
Maria [35] (T5 strain) (Table 1)	1.2526 × 10^4^	567	0.048 (nominal, nonoptimized FBR)
This paper (T5 strain)	1.2526 × 10^4^	532	0.06 and higher (*) (optimized FBR)

(*) By following the same optimal feeding policy, a higher productivity can be obtained for larger batch times (not presented here).

## Data Availability

Restrictions apply to the availability of the experimental data. These data were obtained from a third party (see the acknowledgement) and are available from the authors of the works [70,71,72,73,74] with their permission.

## Nomenclature

$c_{i}$	Species (i) concentration
$c_{x}$	Biomass concentration
$c_{GLC}^{feed}$	Glucose feeding solution concentration
$c_{glc,j}^{feed}$	Glucose feeding solution concentration over the time-arc “J”
$c_{glc,0}^{ext}=c_{GLC}^{ext}(t=0)$	Initial glucose concentration in the bioreactor
$c_{GLC}^{ext}$	Glucose concentration in the bulk phase
$F_{L}$	Liquid feed flow rate in the bioreactor
***k***, $k_{j}$, $K_{j}$*, K, n,* $V_{2m}$, $V_{4m}$, $r_{j}^{\max}$, $a_{x}$, $b_{x}$, $N_{x}$, $r_{uptake}^{\max}$, $K_{PTS,a1}$, $K_{PTS,a2}$, $K_{PTS,a3}$, $V_{2m}$ *g*,, $K_{R}^{amp}$, $K_{T}^{atp}$, $\mu_{T}\;$, $a_{T}$, $b_{T}$, $N_{T}$, etc.-	Reaction rates and/or equilibrium constants of the kinetic model
$r_{i}$	Species (i) reaction rate
t, $t_{{}_{f}}$	Time, batch time
$V_{1}\;-\;V_{6}$	Metabolic fluxes in the glycolysis (Table 2 and Table 3, Figure 3)
$V_{L}$	Liquid volume in the bioreactor
$y_{tca}\;,\;y_{trp}$	Stoichiometric coefficients
**Greeks**	
α, β, γ, δ	Reaction rate constants
μ	Cell content dilution rate, that is $\ln(2)/t_{c}$, where $t_{c}$ denotes the cell cycle
Ω	FBR optimization objective function, Equation (10)
$\rho_{x}$	Biomass density
**Subscripts**	
0,o	Initial
cell	Referring to the cell (inside)
ext	External to cell (i.e., in the bulk phase)
f	Final
inlet	In the feed
x	Biomass
**Abbreviations**	
13dpg, pgp	1,3-Diphosphoglycerate
3pg	3-Phosphoglycerate
2pg	2-Phosphoglycerate
AA	Amino acid
Accoa, acetyl-CoA	Acetyl-coenzyme A
AC	acetate
ADP, adp	Adenosine diphosphate
*AK-ase*	Adenylate kinase
ALE	Adaptive laboratory evolution
AMP, amp	Adenosine monophosphate
ATP, atp	Adenosine triphosphate
*ATP-ase*	ATP monophosphatase
CCM	Central carbon metabolism
CIT	Citrate
CSTR	Continuously stirred tank reactor
DO	Dissolved oxygen
DW	Dry mass
E	Enzyme anthranilate synthase in TRP synthesis model
ETOH	Ethanol
ext	External to the cell (i.e., in the bulk phase)
FBR	Fed-batch bioreactor
FDP, fdp	Fructose-1,6-biphosphate
F6P, f6p	Fructose-6-phosphate
GalP/Glk	Galactose permease/glucokinase
G3P, g3p, GAP, gap, 3PG, 3pg	Glyceraldehyde-3-phosphate
2PG, 2pg	2-Phosphoglycerate
G6P, g6p	Glucose-6-phosphate
GLC, glc	Glucose
Glc(ex), [GLC]ext	Glucose in the environment (bulk phase)
GMO	Genetically modified microorganisms
GRC	Genetic regulatory circuits
*HK-ase*	Hexokinase
JWS	Silicon Cell project of Olivier and Snoep [55]
LAC, lac	Lactate
Max (x)	Maxim of (x)
MMA	The adaptive random optimization algorithm of Maria [93]; Mihail and Maria [99]
mRNA	Tryptophan messenger ribonucleic acid during its encoding gene dynamic transcription and translation
NAD(P)H	Nicotinamide adenine dinucleotide (phosphate) reduced
NLP	Nonlinear programming
ODE	Ordinary differential equations set
OR	The complex between O and R (aporepressor of the TRP gene)
OT	The total TRP operon
P, Pi	Phosphoric acid
PEP, pep	Phosphoenolpyruvate
13DPG=PGP	1,3-Diphosphoglycerate
*PFK-ase*	Phosphofructokinase
PK-ase	Pyruvate kinase
PTS	Phosphotransferase or the phosphoenolpyruvate–glucose phosphotransferase system
PYR, pyr	Pyruvate
QSS	Quasi-steady state
R5P	Ribose 5-phosphate
mRNA	Messenger ribonucleic acid
SUCC, suc	Succinate
TCA, tca	Tricarboxylic acid cycle
TF	Gene expression transcription factors
TRP, Trp, trp	Tryptophan
X	Biomass
Wt.	Weight
[x]	Concentration of species x
